# Synthesis and characterization of diacylgermanes: persistent derivatives with superior photoreactivity†

**DOI:** 10.1039/d1dt02091a

**Published:** 2021-08-05

**Authors:** Sabrina D. Püschmann, Philipp Frühwirt, Stefanie M. Müller, Stefan H. Wagner, Ana Torvisco, Roland C. Fischer, Anne-Marie Kelterer, Thomas Griesser, Georg Gescheidt, Michael Haas

**Affiliations:** Institute of Inorganic Chemistry, Technical University Graz Stremayrgasse 9/IV 8010 Graz Austria michael.haas@tugraz.at; Institute of Physical and Theoretical Chemistry, Technical University Graz Stremayrgasse 9/II 8010 Graz Austria; Institute of Chemistry of Polymeric Materials, Montanuniversitaet Leoben Otto-Gloeckelstrasse 2 A-8700 Leoben Austria

## Abstract

Acylgermanes are known as highly efficient photoinitiators. In this contribution, we present the synthesis of new diacylgermanes **4a–e***via* a multiple silyl abstraction methodology. The method outperforms the state-of-the-art approach (Corey–Seebach reaction) towards diacylgermanes in terms of group tolerance and toxicity of reagents. Moreover, these compounds are decorated with bulky mesityl groups in order to improve their storage stability. The isolated diacylgermanes were characterized by multinuclear NMR-, UV-Vis spectroscopy and X-ray crystallography, as well as photolysis experiments (photobleaching) and photo-DSC measurements (photopolymerization behavior). Upon irradiation with an LED emitting at 385 nm, all compounds except for **4a** and **4c** bleach efficiently with quantum yields above 0.6. Due to their broad absorption bands, the compounds can be also bleached with blue light (470 nm), where especially **4e** bleaches more efficiently than Ivocerin®.

## Abstract 2

A series of novel diacylgermanes with bulky mesityl groups by the multiple silyl abstraction methodology, avoiding the Corey–Seebach reaction were synthesized.

## Graphical abstract

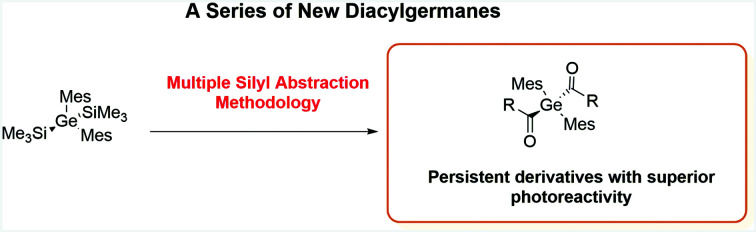

## Introduction

State-of-the-art visible light initiators for photo-induced free radical polymerization are used for a variety of different applications such as 3D-printing,^1^ tissue engineering,^2^ coatings^3^ and dental applications.^4^ The vast majority of these state-of-the-art initiators decompose into radical species *via* Norrish type I (α-cleavage). Modern Norrish type I initiators absorb light above 400 nm *e.g.* acylphosphines,^5^ acylstannanes^6^ and acylgermanes.^7–10^

**Chart 1:**
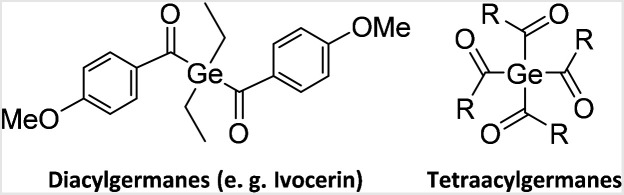
The state-of-the-art germanium-based photoinitiators.

Especially the latter were intensively investigated in the last two decades, which boosted the number of available synthetic pathways towards acylgermanes significantly. However, di(4-methoxybenzoyl)diethylgermane (Ivocerin®), a commercially available diacylgermane, is synthesized *via* a complex procedure, which relies on a Corey–Seebach reaction followed by column chromatography and consequently results in the high costs of this PI (Chart 1).^11^ Another disadvantage is the inefficient curing depth at wavelengths above 500 nm.^7^ Our group has introduced a new one-pot synthetic protocol providing tetraacylgermanes Ge[C(O)R]_4_ (R = aryl) in high yields (Chart 1).^8^ However, most implemented acylgermanes as initiators have stability issues in various solvents limiting the field of applications.

Here, we introduce an easy to perform synthetic pathway towards diacylgermanes **4a–f** with bulky mesityl groups, avoiding the Corey–Seebach reaction. The introduction of these mesityl groups leads to an increased stability in comparison to the state-of-the-art germanium based photoinitiators. Moreover, the broad absorption band of these compounds lead to a good applicability in the field of photopolymerization.

## Results and discussion

### Synthetic procedures

**Scheme 1:**
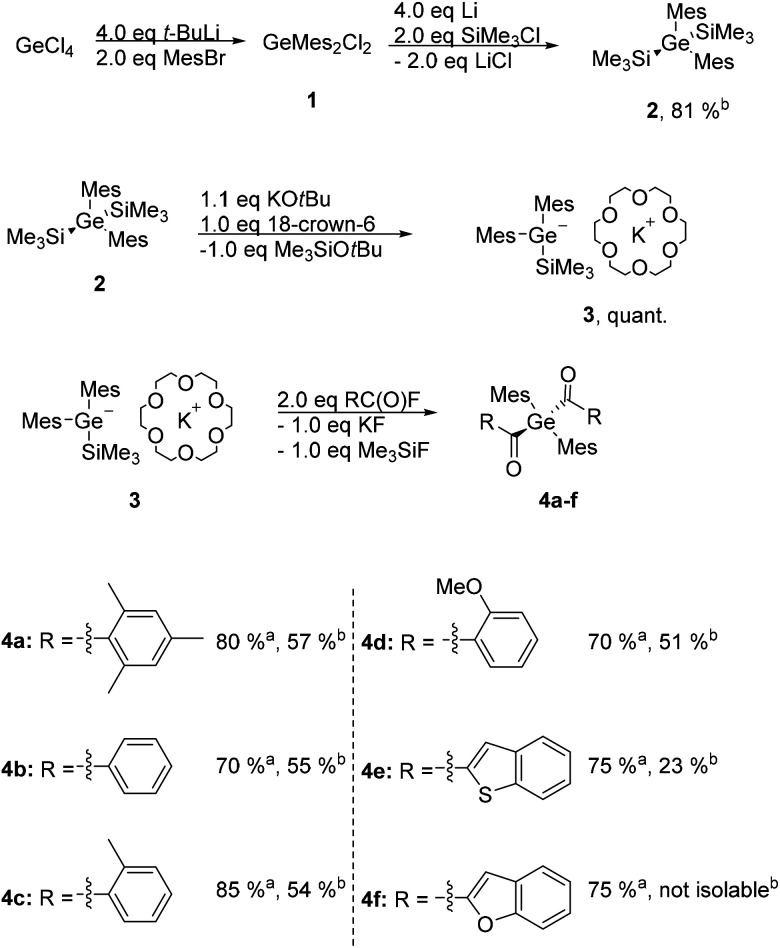
Synthetic protocol towards diacylgermanes **4a–f**. ^*a*^ Yield determined by ^1^H NMR analysis. ^*b*^ Isolated yield.

The entry into this chemistry is provided by the easy to perform synthetic protocol towards dimesityldi(trimethylsilyl)germane **2**. Subsequently **2** was reacted with equimolar amounts of KO*t*Bu and 18-crown-6 generating the germanide **3** as crucial intermediate. On the one hand the addition of 18-crown-6 is necessary to stabilize the anion and on the other hand the usage of crown ether circumvents the introduction of sulfur protecting groups and therefore is more sustainable. Analytical and spectroscopic data clearly support the structure of **3** (for details consult the Experimental section and the ESI†). This germanide was added to the respective twofold excess of acid fluoride *in situ* yielding the desired diacylgermanes **4a–e** in good yields (see Scheme 1). Analytical and spectroscopic data that support the structural assignment are given in the Experimental section, together with experimental details. Particularly striking is the possible synthesis and isolation of compound **4e**, where a benzothiophene group is substituted at the carbonyl moiety, as the introduction of heterocyclic groups is not possible with the Corey–Seebach reaction. Compound **4f** was also formed with this protocol, unfortunately due to the instability with silica gel it was not isolable.

### UV-Vis spectroscopy

**Fig. 1:**
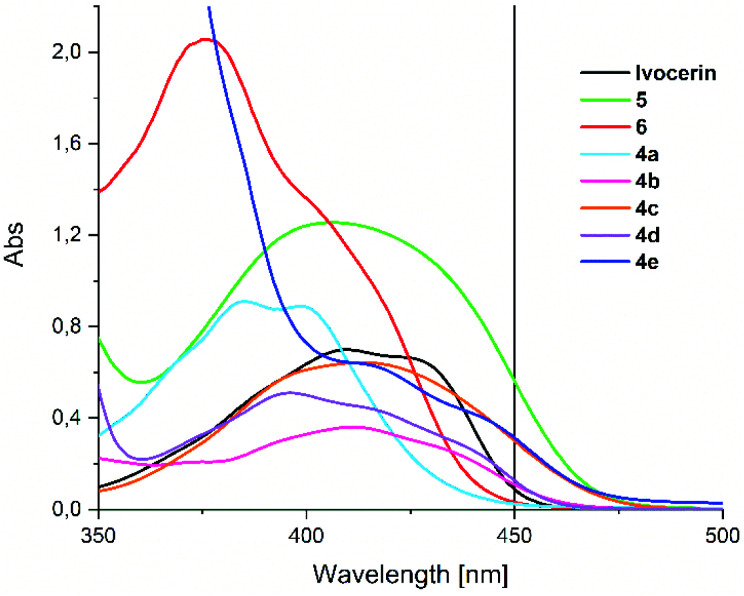
Absorption spectrum of synthesized compounds **4a–e** compared with the commercially available Ivocerin®, tetra(*o*-toluoyl)germane (5) and tetra(mesitoyl)-germane (6) with a concentration of 1 × 10^−3^ M in chloroform.

The broad absorption band of compounds **4a–e** are centered at around 410 nm (see Fig. 1), which can be allocated to the n–π* transition. This band is responsible for the photo-induced cleavage of the Ge–C bond. In comparison to Ivocerin® these new diacylgermanes show broader absorption bands, which results in a bathochromic shift of their absorption edge. Consequently, **4b–e** absorb light above 450 nm whereas the commercial PI, Ivocerin®, has a weak performance at this wavelength. In comparison to tetra(*o*-toluoyl)germane **5** and tetra(mesitoyl)germane **6** as representative examples of tetraacylgermanes, **4a–e** have similar absorption edges, however lower extinction coefficients, which is due to the presence of only two chromophore groups at the diacylgermanes, compared to the four chromophores in the case of tetraacylgermanes.

### DFT calculation

Based on the X-ray data, we have optimized the geometries of **4a–4e** by DFT. Basically, the calculated geometries correspond with the experimental data. For **4a**, an additional, more stable conformer (*E*_rel_ = 8.2 kJ mol^−1^) was found. However, the electronic transitions calculated for the latter isomer were essentially identical with those computed for that corresponding to the X-ray structure. The vertical excitation energies (TDDFT) and are presented in the ESI (Tables S3 and S4†).

**Fig. 2:**
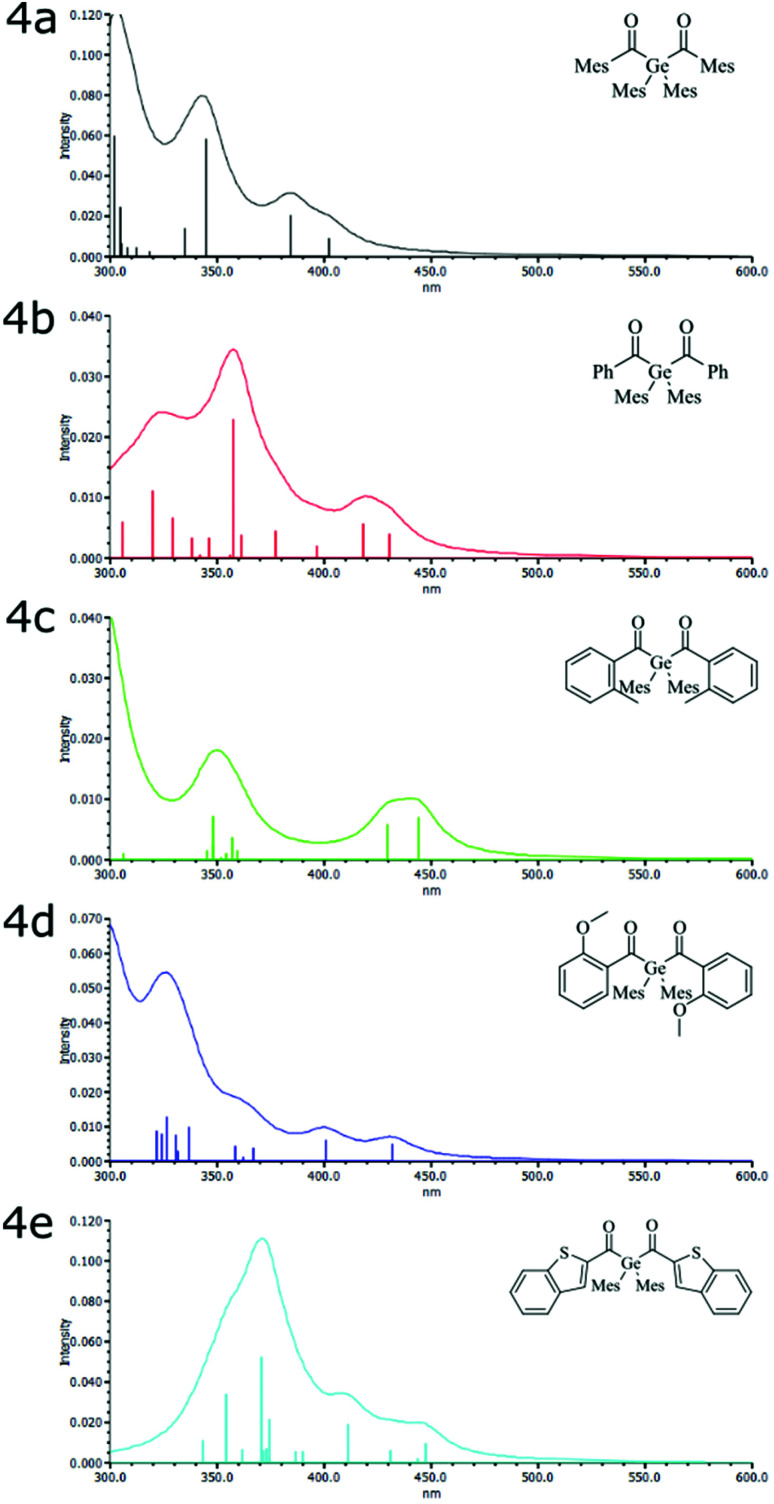
Simulated spectrum of **4a–e** together with the vertical transitions (vertical lines).

The TDDFT calculated excitation spectra of **4a–e** agree well with the experimental UV-Vis spectra (Fig. 2). The first absorption band at *ca.* 420 nm consists of two vertical excitations, HOMO → LUMO and HOMO → LUMO+1. Both transitions are n–π* transitions. The HOMO is localized on both oxygen lone pairs of the C=O groups. LUMO and LUMO+1 are both linear combinations of the C=O π-orbitals with contributions of the neighboring aromatic system. The influence of substitution on orbital energies and localization can be explained by considering the electronic donating (EDG) effects of the substituents. The methyl group is inductive electronic donating and the methoxy group is mesomeric electronic donating. Consequently, the presence of EDG groups (Me in **4a** and **4c** and OMe in **4d**) increase the electron density of the HOMO orbital on the aromatic systems. Additionally, both EDG groups reduce the HOMO–LUMO-gap slightly compared to the unsubstituted **4b** (Table S4†), and, thus, cause a bathochromic shift (see Table S3† and Fig. 2).

The extension of the π system in **4e** by the annelated thiophene also leads to a red-shift compared with **4a–d**. For **4e** the four calculated transitions between 411 and 447 nm are in good consent with the rather broad absorptions between *ca.* 400 and 470 nm. Whereas the lines at 411, 431, and 447 nm possess two dominating components contributions, that at 443 is dominated by the HOMO–LUMO transition (Table S3†).

**Fig. 3:**
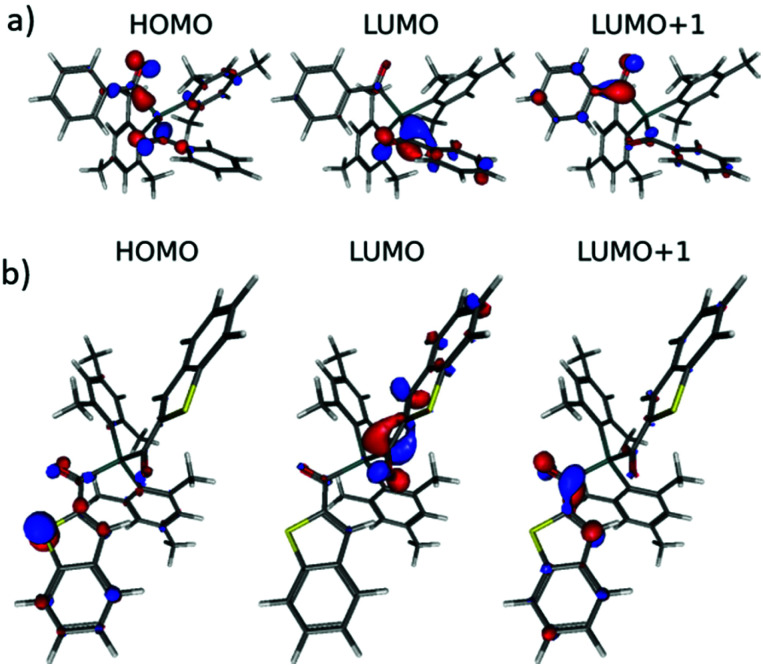
(a) Relevant orbitals for the first two vertical excitations of **4b**. (b) Relevant orbitals for the first two vertical excitations of **4e**.

Fig. 3 shows the relevant orbitals for the first two vertical excitations of **4b** and **4e**. For all other compounds the relevant orbitals can be found in the ESI (Fig. S34†).

In particular, the dihedral angle between the aromatic substituent and the adjacent acyl group directs the electronic properties of the diacylgermanes. The bulky mesityl group in **4a** causes an almost perpendicular arrangement of the C=O group and the π-plane of the mesityl substituent (dihedral angle = 75.8°) whereas the π-system and the C=O group are almost coplanar in **4e** (3.28°) and **4c** (21.3°) > **4d** (16.36°) > **4b** (12.4°) being “intermediate” cases.

Consequently, in **4a**, the electron density is almost entirely localized at the acyl group (Fig. S34†). Solely, HOMO−1 indicates distinguishable coefficients at one mesityl group. For **4c–4e** with smaller dihedral angles delocalization is more pronounced rationalizing the red shifts in the UV-VIS spectra.

### Photobleaching

Efficient curing of photoinitiator/monomer mixtures is crucial for applications like dental restoration, therefore steady-state photolysis experiments were performed to assess the photobleaching behavior of the compounds. To that end, degassed solutions of **4a–e** in a 1/1 (v/v) mixture of toluene/methyl methacrylate (with absorbance of about 0.7 at 385 nm) were irradiated with two different low power LEDs with emission maxima at about 385 and 470 nm (LED385 and LED470; described in the Experimental section of the ESI† in more detail) – emission wavelengths, which are also found in commercial dental lamps.^12^

**Fig. 4:**
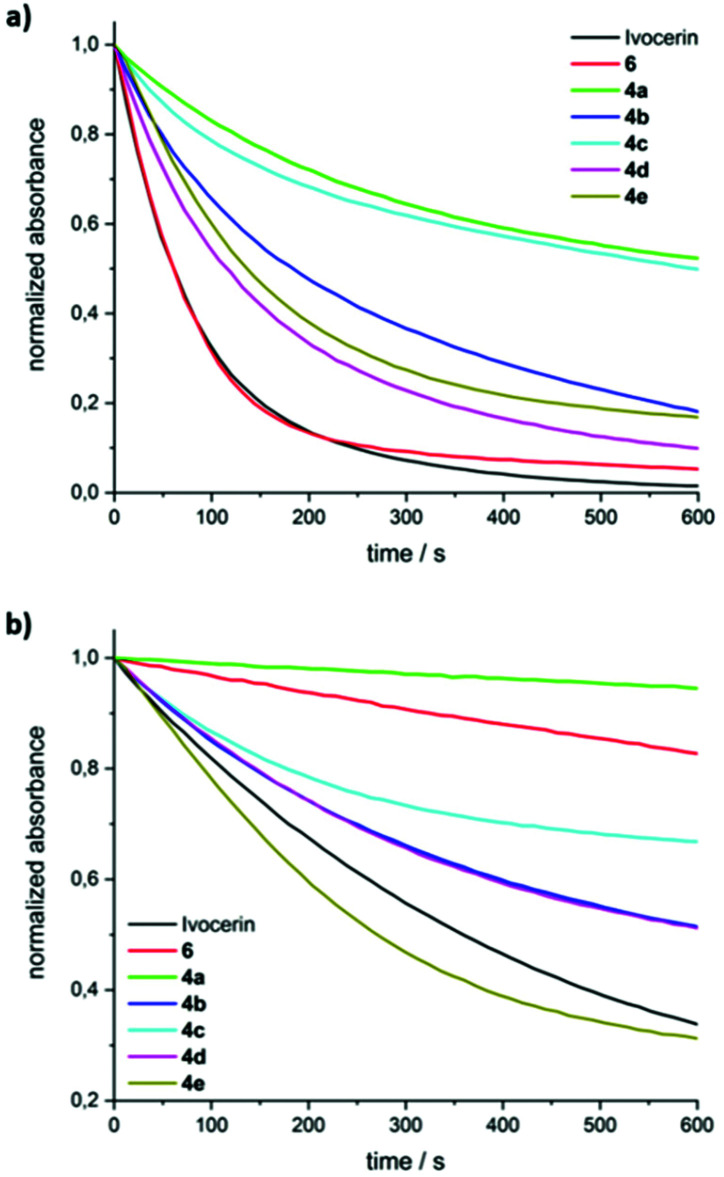
Steady-state photolysis of **4a–e** with (a) LED385, (b) LED470 in toluene/MMA (1/1 v/v). The absorbance traces are normalized to the initial absorptions at the observation wavelengths (maxima of n/σ–π* transitions; **4a**: 387 nm, **4b**: 413 nm, **4c**: 413 nm, **4d**: 399 nm, **4e**: 419 nm). Data for Ivocerin® and tetramesitoylgermane (6) were taken from ref. 10.

Fig. 4 shows the time traces of the normalized absorbances when irradiating with LED385 or LED470. It can be seen that **4a** and **4c** bleach least efficiently, whereas **4d** is almost comparable to Ivocerin® or to **6**. From an exponential fit of the concentration traces, the quantum yields of decomposition can be determined using the procedure by Stadler *et al.*^13^ The compounds **4a** and **4c** feature the smallest quantum yields (0.20 and 0.49, respectively), while the quantum yields of the other compounds are above 0.62 (see Table 1). For compound **4d** a higher quantum yield comparable to the one of Ivocerin® (*ϕ* = 0.83^14^) is obtained. We ascribe this to the electronic properties of the methoxy groups having a compatible effect when residing in either *o*- or *p*-position of the benzoyl substituents. Despite the fact, that **4c** and **4e** show nearly identical extinctions above 450 nm (see Fig. 1), photobleaching of **4e** with the blue LED emitting at 470 nm is more efficient due to its higher quantum yield of decomposition and even faster than Ivocerin® (see Fig. 4b).

**Table 1:** Wavelength of n/σ–π* absorption maxima and extinction coefficients (in toluene/MMA 1/1 (v/v)) and determined quantum yields of **4a–e**

Compound	*λ*_max,exp_ [nm]	*ε* [M^−1^ cm^−1^] at *λ*_max,exp_	*Φ* (385 mm)
**4a**	387	903	0.20 ± 0.01
**4b**	413	510	0.68 ± 0.02
**4c**	413	624	0.49 ± 0.02
**4d**	399	495	0.83 ± 0.02
**4e**	419	606	0.62 ± 0.01

### NMR spectroscopy

NMR spectra and detailed characterization of **4a–f** are provided in the ESI.† All compounds show very similar ^13^C chemical shifts for the carbonyl carbon between 219.47 ppm and 240.67 ppm, which is characteristic for the carbonyl groups directly linked to the germanium atom.

### Storage stability tests

**Fig. 5:**
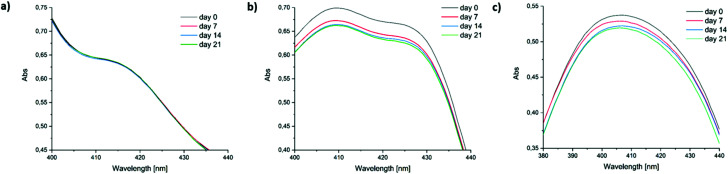
Comparison of the long-term stability in solution (1 × 10^−3^ M in chloroform) detected by UV-Vis spectroscopy. Zoomed in part of the UV-Vis spectra of (a) compound **4e**, (b) Ivocerin® and (c) 5.

**Fig. 6:**
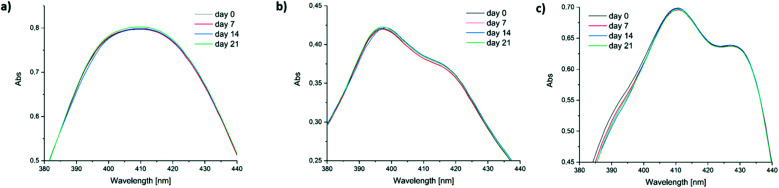
Comparison of the long-term stability in solution (1 × 10^−3^ M in MMA) detected by UV-Vis spectroscopy. (a) **4c**, (b) **4d** and (c) Ivocerin®.

Low long-term stability in various solvents (such as chloroform, benzene or toluene) is a drawback of Ivocerin® and teraacylgermanes limiting the field of applications. By introducing bulky mesityl groups at the germanium atom the storage stability was drastically improved. UV-Vis spectroscopy as well as NMR spectroscopy were used to determine the storage stability of Ivocerin®, tetra(*o*-toluoyl)germane **5** and compounds **4a–e**. For the UV-Vis method, all acylgermanes were measured in chloroform with a concentration of 1 × 10^−3^ M and the spectra were recorded once a week over a period of 21 days. To confirm the stability in MMA, UV-Vis spectra of Ivocerin®, reference compound **5**, compound **4c** and **4d** were recorded once a week over a period of 21 days with a concentration of 1 × 10^−3^ M. In case of the NMR method, the measurements were performed in degassed CDCl_3_ and C_6_D_6_ (0.01 M in 0.5 mL). Once a week over a period of 21 days a spectrum of those were recorded. During these long-term tests, the NMR tubes, as well as the brown glass volumetric flask for the UV-Vis measurements were sealed with parafilm and stored in the dark. As representative example, compound **4e**, as well as the spectra of Ivocerin® and tetra(*o*-toluoyl)germane for comparison, are shown in Fig. 5 (UV-Vis data) and Fig. 8 (NMR data, left: in C_6_D_6_, right: in CDCl_3_). The measurements in MMA are shown in Fig. 6. All other spectra can be found in the ESI.†

As shown in Fig. 5, the newly synthesized compound **4e** is stable and even after 21 days no degradation is observed. In contrast to that the absorption of Ivocerin® and **5** slightly decrease (see Fig. 5b and c). Moreover, we could confirm by our stability tests that all investigated diacylgermanes are stable in MMA (Fig. 6a–c).

To conclude, the commercial PIs degrade in solution, in particular in chloroform as well as in benzene, whereas the new synthesized compounds **4a–e** are significantly more stable and show no degradation. In monomers, like in MMA, diacylgermanes are stable over time.

The same statement can be made after measuring the NMR spectra of all compounds. The newly synthesized compounds (*e.g.***4e**) do not show any degradation. Even after 21 days, the spectra remain unaffected (see Fig. 8a and b). On the opposite, Ivocerin® and **5** already show degradation after 7 days. During the course of this experiments the degradation product, the aldehyde, get more and more dominant, until only the degradation product is present (Table 2 and Fig. 8). Although the acute toxicity of aldehydes is low, the exposure to aldehydes causes irritation of the skin, eyes and mucous membranes of the respiratory passage. As our new presented initiators **4a–e** do not show any formation of these degradation product, we think that this is an important step forward in this research field.

**Table 2:** Formed aldehyde [%] as degradation product during the stability test monitored by ^1^H analysis of Ivocerin® and compound **5** after **7**, **14** and **21** days in benzene and chloroform. In case of **4e** 0% aldehyde is formed over the measured time

Compound	Solvent	Days	Aldehyde formation [%]
Ivocerin®	C_6_D_6_	7	15
		14	28
		21	50
	CDCl_3_	7	18
		14	32
		21	100

**5**	C_6_D_6_	7	25
		14	62
		21	100
	CDCl_3_	7	44
		14	73
		21	100

### X-Ray crystallography

**Fig. 7:**
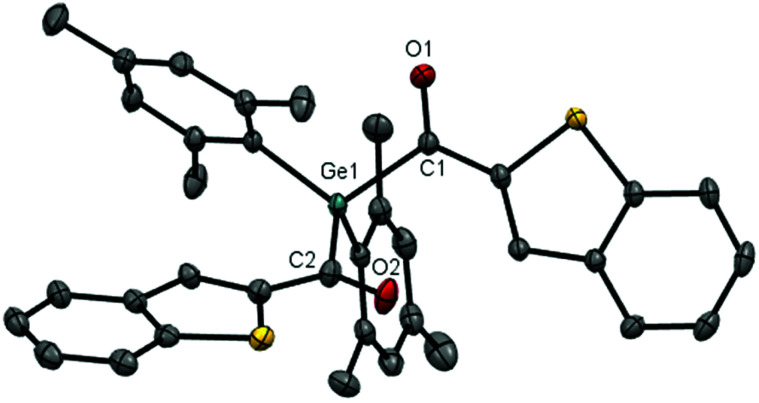
ORTEP representation of **4e**. Thermal ellipsoids are depicted at the 50% probability level. Hydrogen atoms are omitted for clarity. The torsion angle (mean value) between the C=O group and the aromatic ring plane of the thiofuran group is 1.72°.

Crystals suitable for single-crystal XRD were obtained for compounds **4a–e**. Compound **4e** is shown as representative example (Fig. 7), the other structures can be found in the ESI.† The torsion angle between the C=O bond and the aromatic plane vary a lot among the different substituents. The evaluated data of the bond lengths are slightly elongated compared to the average Ge–C bond (1.97 Å)^15^ and the average C=O bond (1.19 Å) (Table 3).^16^

**Table 3:** Mean bond lengths *d* [Å] and torsion angles between the C=O group and the aromatic ring plane [°] of compounds **4a–e**

Compound	*d*_Ge–C_	*d*_C=O_	∠O=C–R
**4a**	2.060	1.213	68.45
**4b**	2.041	1.219	7.95
**4c**	2.044	1.217	25.54
**4d**	2.033	1.217	13.19
**4e**	2.032	1.223	1.72

### Photo-DSC measurements

Photo-DSC is a versatile method to evaluate the performance of PIs in polymerizable resins. One single measurement can give information about the reaction kinetics (time to reach the maximum heat flow (*t*_max_), maximum rate of polymerization (*R*_p,max_), time to reach 95% of final conversion (*t*_95%_)) and the double bond conversion (DBC, calculated from the overall reaction enthalpy Δ*H* (peak area) and the theoretical heat of polymerization (Δ*H*_0,p_)).

The photopolymerization experiments were conducted in 1,6-hexanediol diacrylate (HDDA) as model monomer system (for further details consult the ESI†). Besides the synthesized PIs (*i.e.***4a–4e**), the polymerization behavior of Ivocerin® and **5** was determined as reference.

The PI performance was analyzed at (1) equal molar PI (0.30 mol%) as well as (2) equal photo-cleavable group (PCG) concentration (0.15 mol% for **5**; 0.30 mol% for **4a–4e** and Ivocerin®), respectively. In general, the synthesized PIs provide kinetics and a DBC comparable to the reference compounds. In more detail, **4b** and **4d** show a slightly faster polymerization initiation than the other synthesized PIs and can be compared in their reactivity with **5** at the same PI concentration. However, measured at similar PCG concentration, they show a slightly slower turnover than Ivocerin®, but higher double bond conversion than both Ivocerin® and **5**. This correlates to the quantum yields measured at 385 nm, where **4b** and **4d** show the highest values (Fig. 9).

**Fig. 8:**
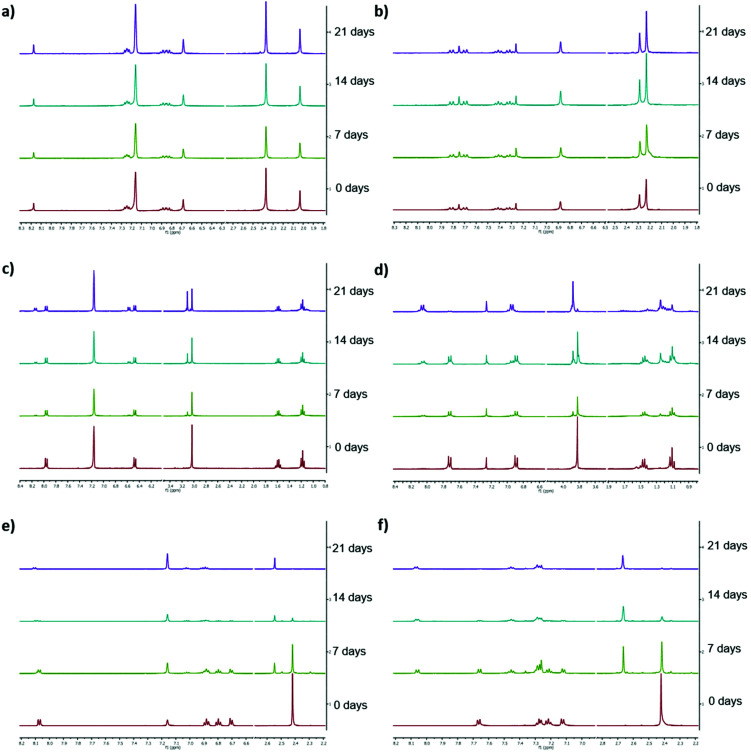
Representation of the stacked ^1^H NMR spectra (300 MHz) of the respective compound (0.01 M in 0.5 mL) in the corresponding degassed solvent, left: in C_6_D_6_ (61.7 ppm H_2_O) right: in CDCl_3_ (26 ppm H_2_O). (a) shows compound **4e** in C_6_D_6_, (b) compound **4e** in CDCl_3_, (c) Ivocerin® in C_6_D_6_, (d) Ivocerin® in CDCl_3_, (e) **5** in C_6_D_6_ and (f) **5** in CDCl_3_.

**Fig. 9:**
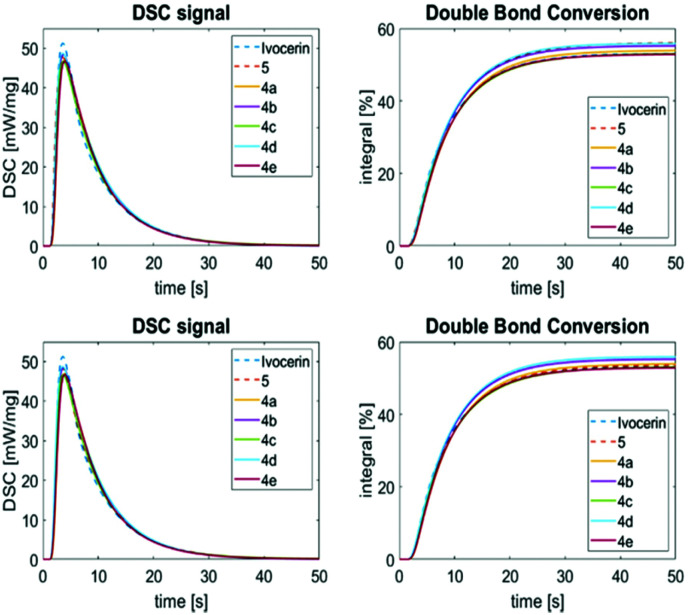
Photo-DSC (left) and conversion plots (right) for the photopolymerization of HDDA with 0.3 mol% PI (top) and equal PCG concentration (bottom).

## Experimental

### General considerations

All synthetic steps were performed under inert conditions using standard Schlenk techniques. Solvents were dried with a column purification system.^17^ Commercial tetrachlorogermane (GeCl_4_), *tert*-butyllithium (*t*-BuLi), mesitylbromide (MesBr), chloro(trimethyl)silane (SiMe_3_Cl), and KO*t*Bu were used without further purification. The used acid fluorides were produced according to the corresponding literature.^18^^1^H (299.95 MHz) and ^13^C (75.43 MHz) NMR spectra were recorded with a Varian INOVA 300 spectrometer in CDCl_3_ or C_6_D_6_ solution and were referenced *versus* TMS by using the internal ^2^H lock signal of the solvent. UV-Vis spectra were recorded with an Agilent Cary 60 UV-Vis spectrometer. Mass spectra were acquired with a Q-TOF Premier from Waters, Manchester, England. The original ESI source of the instrument was replaced by a standard LIFDI source from Linden CMS, Weyhe, Germany. IR spectra were recorded with a Brucker ALPHA. Melting points were determined by a Stuart automatic melting point (SMP50).

### Synthesis of dichlorodimesitylgermane (**1**)

A 3.0 L flask was charged with MesBr (52 mL, 339 mmol, 2.00 eq.) and THF (800 mL). The solution was cooled to −78 °C. 400 mL of *t*-Buli (1.70 M, 678 mmol, 4.00 eq.) were added with a dropping funnel and stirred for additional 30 minutes. Afterwards GeCl_4_ (36.4 g, 170 mmol, 1.00 eq.) was dissolved in small amounts of THF and were added to the reaction. After warming up to room temperature, the reaction was stirred overnight. The reaction mixture was transferred into a flask and the solvent was removed under reduced pressure. The solid was extracted with pentane and the solvent was again removed under reduced pressure leading to white crystals (24.4 g, 38%).

^**1**^**H NMR** (300 MHz, CDCl_3_, ppm): *δ* 6.81 (s, 4H, Mes–*H*), 2.28 (s, 12H, Mes–*o*C*H*_3_), 2.26 (s, 6H, Mes–*p*C*H*_3_).

### Synthesis of dimesityldi(trimethylsilyl)germane (**2**)

A 500 mL 3-neck flask with a dropping funnel and a reflux condenser was charged with THF (90 mL) and Li (1.70 g, 249 mmol, 4.00 eq.), which was cooled to −70 °C. Trimethylchlorosilane (13.3 mL, 104 mmol, 3.35 eq.) was added to the solution and afterwards compound **1** (11.9 g, 31.1 mmol, 1.00 eq.) was dissolved in THF (90 mL) and was added over one hour. The reaction was stirred for further 2 h at −70 °C. The cooling was removed and the reaction was stirred over night at room temperature. After aqueous workup with saturated NH_4_Cl, the phases were separated and the aqueous phase was extracted three times with diethyl ether. The organic layer was dried over Na_2_SO_4_, filtered and the solvent was removed under reduced pressure. The crude product was recrystallized from acetone at −30 °C to yield 11.5 g (81%).

^**1**^**H NMR** (300 MHz, CDCl_3_, ppm): *δ* 6.76 (s, 4H, Mes–*H*), 2.23 (s, 6H, Mes–*p*C*H*_3_), 2.17 (s, 12H, Mes–*o*C*H*_3_), 0.21 (s, 18H, Si–(C*H*_3_)_3_).

### Synthesis of dimesityldimesitoylgermane (**4a**)

Mes_2_Ge(SiMe_3_)_2_**2** (2.00 g, 4.37 mmol, 1.00 eq.), 18-crown-6 (1.27 g, 4.81 mmol, 1.10 eq.) and KO*t*Bu (0.54 g, 4.81 mmol, 1.10 eq.) were dissolved 25 mL benzene and stirred for 1 h at room temperature. A second flask was charged with 2,4,6-trimethylbenzoyl fluoride (1.45 g, 8.75 mmol, 2.00 eq.) in toluene (10 mL) and cooled to 0 °C. The germanide **3** was added dropwise to the fluoride solution. The cooling was removed, and the reaction mixture was allowed to warm to room temperature and stirred overnight. After aqueous workup with saturated NH_4_Cl, the phases were separated and the aqueous phase was extracted three times with DCM. The organic layer was dried over Na_2_SO_4_, filtered with silica gel to remove the crown ether, and the solvent was removed under reduced pressure. The crude product was recrystallized from pentane at −70 °C to yield 1.51 g (57%) of pure slightly yellow crystals.

**M.p.** 183–186 °C; **UV-Vis** (chloroform): *λ* = 385 and 399 nm, *ε* = 924 and 902 L mol^−1^ cm^−1^; **IR**: *v*[cm^−1^] = 1636, 1603 (m, *ν*C=O); **elemental analysis** (%) calcd for C_38_H_44_GeO_2_: C 75.39, H 7.33; found: C 75.31, H 7.34; ^**1**^**H NMR** (300 MHz, CDCl_3_, ppm): *δ* 6.59 (s, 4H, C(O)Mes–*H*), 6.51 (s, 4H, Mes–*H*), 2.12 (s, 6H, C(O)Mes–*p*C*H*_3_), 2.12 (s, 6H, Mes–*p*C*H*_3_), 1.98 (s, 12H, C(O)Mes–*o*C*H*_3_), 1.91 (s, 12H, Mes–*o*C*H*_3_). ^**13**^**C NMR** (75 MHz, CDCl_3_, ppm): *δ* 240.67 (Ge*C*OMes), 143.99, 142.35, 138.88, 138.73, 135.25, 134.15, 129.32, 128.75, 128.69 (Aryl–*C*), 25.14 (Mes–*oC*H_3_), 21.17 (Mes–*pC*H_3_), 21.05 (Mes–*pC*H_3_), 19.68 (Mes–*oC*H_3_). **HRMS**: calcd for [C_38_H_44_GeO_2_]^+^ (M^+^): 606.2562. Found: 606.3292.

### Synthesis of dimesityldibenzoylgermane (**4b**)

Compound **2** (4.00 g, 8.75 mmol, 1.00 eq.), 18-crown-6 (2.54 g, 9.62 mmol, 1.10 eq.) and KO*t*Bu (1.08 g, 9.62 mmol, 1.10 eq.) were dissolved in benzene (60 mL) and stirred for 1 h at room temperature. The germanide **3** was added dropwise to a solution of benzoyl fluoride (1.90 mL, 17.5 mmol, 2.00 eq.) in toluene (10 mL) at 0 °C. After heating up to room temperature, the orange-red reaction solution was stirred overnight. Followed by aqueous workup with NH_4_Cl solution, extraction with DCM, drying over Na_2_SO_4_ and filtration over silica gel to remove the excess of crown ether. The solvent was removed under reduced pressure. The crude product was further purified by column chromatography (pentane/toluene 1 : 2) and recrystallized from pentane at −70 °C, yielding 2.50 g (55%) of yellow crystals.

**M.p.** 155–157 °C; **UV-Vis** (chloroform): *λ* = 411 and 437 (sh) nm, *ε* = 369 and 241 (sh) L mol^−1^ cm^−1^; **IR**: *v*[cm^−1^] = 1620, 1590, 1574 (m, *ν*C=O); **elemental analysis** (%) calcd for C_32_H_32_GeO_2_: C 73.74, H 6.19; found: C 73.31, H 6.17; ^**1**^**H NMR** (300 MHz, CDCl_3_, ppm): *δ* 7.80–7.77 (d, *J* = 7.3 Hz, 4H, Ph–*H*), 7.44–7.39 (t, *J* = 7.3 Hz, 2H, Ph–*H*), 7.31–7.28 (d, *J* = 7.7 Hz, 4H, Ph–*H*), 6.87 (s, 4H, Mes–*H*), 2.28 (s, 6H, Mes–*p*C*H*_3_), 2.20 (s, 12H, Mes–*o*C*H*_3_). ^**13**^**C NMR** (76 MHz, CDCl_3_, ppm): *δ* 228.15 (Ge*C*OPh), 143.59, 140.61, 139.45, 134.12, 133.04, 129.64, 128.75, 128.35 (Aryl–*C*), 25.11 (Mes–*oC*H_3_), 21.15 (Mes–*pC*H_3_). **HRMS**: calcd for [C_32_H_32_GeO_2_]^+^ (M^+^): 522.1622. Found: 522.2216.

### Synthesis of dimesityldi(*o*-toluoyl)germane (**4c**)

Compound **2** (4.00 g, 8.75 mmol, 1.00 eq.), 18-crown-6 (2.54 g, 9.62 mmol, 1.10 eq.) and KO*t*Bu (1.08 g, 9.62 mmol, 1.10 eq.) were dissolved in benzene (60 mL) and stirred for 1 h at room temperature. A second flask was charged with *o*-toluoyl fluoride (2.43 g, 17.5 mmol, 2.00 eq.) and dissolved in 10 mL toluene. The solution was cooled to 0 °C. After complete addition of the germanide **3** to this solution, aqueous workup with 10% H_2_SO_4_ followed. The aqueous phase was extracted three times with DCM, dried over Na_2_SO_4_, filtrated over silica gel and the solvent was removed under reduced pressure. The crude product was further purified by column chromatography (pentane/toluene 2 : 1) and recrystallization from pentane at −70 °C, pure yellow crystals were isolated (2.59 g, 54%).

**M.p.** 142 °C; **UV-Vis** (chloroform): *λ* = 415 nm, *ε* = 658 L mol^−1^ cm^−1^; **IR**: *v*[cm^−1^] = 1630, 1598, 1563 (m, *ν*C=O); **elemental analysis** (%) calcd for C_34_H_36_GeO_2_: C 74.35, H 6.61; found: C 74.19, H 6.45; ^**1**^**H NMR** (300 MHz, CDCl_3_, ppm): *δ* 7.68–7.66 (d, *J* = 7.6 Hz, 2H, Ph–*H*), 7.20–7.15 (t, *J* = 7.4 Hz, 2H, Ph–*H*), 7.06–7.01 (t, *J* = 7.1 Hz, 4H, Ph–*H*), 6.86 (s, 4H, Mes–*H*), 2.31 (s, 6H, Ph–*o*C*H*_3_), 2.27 (s, 6H, Mes–*p*C*H*_3_), 2.25 (s, 12H, Mes–*o*C*H*_3_). ^**13**^**C NMR** (76 MHz, CDCl_3_, ppm): *δ* 231.25 (Ge*C*O), 143.66, 140.04, 139.22, 137.19, 134.59, 132.60, 132.44, 131.99, 129.61, 129.57, 125.20 (Aryl–*C*), 25.15 (Mes–*oC*H_3_), 25.02 (*o*Tol–*C*H_3_), 21.49 (Mes–*pC*H_3_). **HRMS**: calcd for [C_34_H_36_GeO_2_]^+^ (M^+^): 550.1927. Found: 550.2034.

### Synthesis of dimesityldi(*o*-methoxy)germane (**4d**)

Compound **2** (4.00 g, 8.75 mmol, 1.00 eq.), 18-crown-6 (2.54 g, 9.62 mmol, 1.10 eq.) and KO*t*Bu (1.08 g, 9.62 mmol, 1.10 eq.) were dissolved in benzene (60 mL) and stirred for 1 h at room temperature. In a second flask, *o*-methoxybenzoyl fluoride (2.71 g, 17.5 mmol, 2.00 eq.) was dissolved in toluene (10 mL) and cooled to 0 °C. The germanide **3** was added dropwise to the fluoride and after warming up to room temperature stirred overnight. Followed by aqueous workup with 10% H_2_SO_4_, extraction with dichloromethane, drying over Na_2_SO_4_, filtration over silica gel and removal of the solvent under reduced pressure. The crude product was further purified by column chromatography (pentane/toluene 1 : 3) and recrystallization from pentane at −70 °C, **4d** was isolated as yellow crystals (2.64 g, 52%).

**M.p.** 201–203 °C; **UV-Vis** (chloroform): *λ* = 396, 418 (sh) and 440 (sh) nm, *ε* = 516, 429 (sh) and 265 (sh) L mol^−1^ cm^−1^; **IR**: *v*[cm^−1^] = 1617, 1590 (m, *ν*C=O); **elemental analysis** (%) calcd for C_34_H_36_GeO_4_: C 70.25, H 6.24; found: C 70.02, H 6.10; ^**1**^**H NMR** (300 MHz, CDCl_3_, ppm): *δ* 7.58–7.55 (d, *J* = 7.7 Hz, 2H, Ph–*H*), 7.15–7.10 (t, *J* = 7.8 Hz, 2H, Ph–*H*), 6.82 (s, 4H, Mes–*H*), 6.79 (d, *J* = 7.7 Hz, 2H, Ph–*H*), 6.35–6.62 (d, *J* = 8.3 Hz, 2H, Ph–*H*), 3.16 (s, 6H, OC*H*_3_), 2.26 (s, 12H, Mes–*o*C*H*_3_), 2.24 (s, 6H, Mes–*p*C*H*_3_). ^**13**^**C NMR** (76 MHz, CDCl_3_): *δ* 222.52 (Ge*C*O), 158.49, 144.05, 137.81, 135.71, 134.04, 128.72, 126.96, 120.43, 110.29 (Aryl–*C*), 52.25 (O*C*H_3_), 24.28 (Mes–*oC*H_3_), 21.09 (Mes–*pC*H_3_). **HRMS**: calcd for [C_34_H_36_GeO_4_]^+^ (M^+^): 582.1833. Found: 582.2579.

### Synthesis of dimesityldibenzothiophenegermane (**4e**)

A flask was charged with Mes_2_Ge(SiMe_3_)_2_**2** (1.00 g, 2.18 mmol, 1.00 eq.), 18-crown-6 (0.64 g, 2.40 mmol, 1.10 eq.) and KO*t*Bu (0.27 g, 2.40 mmol, 1.10 eq.). The compounds were dissolved in benzene (20 mL) and stirred for 1 h at room temperature. In a second flask, thiofuran fluoride (0.99 g, 5.47 mmol, 2.50 eq.) was dissolved in 10 mL toluene and cooled to 0 °C. The germanide **3** was added dropwise to the acid fluoride and was allowed to warm up to room temperature. At this temperature, the reaction was stirred overnight. Followed by aqueous workup with saturated NH_4_Cl solution and the aqueous layer was extracted with DCM. The combined organic phases were dried over Na_2_SO_4_, filtered over silica gel and the solvent was removed under reduced pressure. The crude product was further purified by column chromatography (pentane/toluene 1 + 2) and recrystallization from pentane at −70 °C, yielding in 0.25 g (23%) of yellow crystals.

**M.p.** 171–174 °C; **UV-Vis** (chloroform): *λ* = 371 (sh), 414 (sh), 441 (sh) nm, *ε* = 1990 (sh), 636 (sh) and 403 (sh) L mol^−1^ cm^−1^; **IR**: *v*[cm^−1^] = 1602, 1591 (m, *ν*C=O); **elemental analysis** (%) calcd for C_36_H_32_GeO_2_S_2_: C 68.27, H 5.09, S 10.12; found: C 68.31, H 5.08, S 10.09; ^**1**^**H NMR** (300 MHz, CDCl_3_) *δ* 7.82–7.80 (d, *J* = 8.1 Hz, 2H, Aryl–*H*), 7.75 (s, 2H, Aryl–*H*), 7.71–7.68 (d, *J* = 7.8 Hz, 2H, Aryl–*H*), 7.44–7.38 (t, *J* = 7.6 Hz, 2H, Aryl–*H*), 7.34–7.29 (t, *J* = 7.5 Hz, 2H, Aryl–*H*), 6.88 (s, 4H, Mes–*H*), 2.29 (s, 6H, Mes–*p*C*H*_3_), 2.23 (s, 12H, Mes–*o*C*H*_3_). ^**13**^**C NMR** (76 MHz, CDCl_3_) *δ* 219.47 (Ge*C*O), 148.34, 143.98, 142.22, 140.05, 139.37, 133.13, 129.89, 127.84, 126.89, 124.78, 123.14 (Aryl–*C*), 25.21 (Mes–*oC*H_3_), 21.22 (Mes–*pC*H_3_). **HRMS**: calcd for [C_34_H_36_GeO_4_]^+^ (M^+^): 634.1062. Found: 634.1927.

### Synthesis of dimesityldibenzofurangermane (**4f**)

Compound **2** (0.50 g, 1.09 mmol, 1.00 eq.) was dissolved in benzene (15 mL). KO*t*Bu (0.14 g, 1.20 mmol, 1.10 eq.) and 18-crown-6 (0.32 g, 1.20 mmol, 1.10 eq.) were added and the reaction was stirred for 1 h at room temperature. A second flask was charged with benzofuran fluoride (0.45 g, 2.73 mmol, 2.50 eq.) and was dissolved in toluene (9 mL). The germanide **3** was added to the fluoride solution at 0 °C and after full addition the solution was allowed to warm up to room temperature. At this temperature, the reaction was stirred overnight. A portion was separated and the solvent was removed, which was then dissolved in CDCl_3_. A NMR spectrum was measured. Followed by aqueous workup with saturated NH_4_Cl solution and the aqueous layer was extracted with DCM. The combined organic phases were dried over Na_2_SO_4_, filtered and the solvent was removed under reduced pressure. The purification by either crystallization (normal atmosphere and inert atmosphere) or by column chromatography was not successful. Therefore, clean **4f** could not be isolated.

^**1**^**H NMR** (300 MHz, CDCl_3_) *δ*^1^H NMR (300 MHz, CDCl_3_) *δ* 7.57–7.50 (dd, *J* = 17.7, 7.2 Hz, 4H, Aryl–*H*), 7.34–7.33 (m, 4H, Aryl–*H*), 7.23–7.21 (d, *J* = 5.9 Hz, 2H, Aryl–*H*), 6.86 (s, 4H, Mes–*H*), 2.34 (s, 6H, Mes–*p*C*H*_3_), 2.25 (s, 12H, Mes–*o*C*H*_3_).

## Conclusions

To conclude, we were able to synthesize a variety of substituted diacylgermanes **4a–e** with bulky mesityl groups by the multiple silyl abstraction methodology, avoiding the Corey–Seebach reaction. Moreover, these compounds show excellent stabilities in solvents, which was determined by long-term stability tests in chloroform, benzene and MMA *via* UV-Vis spectroscopy and in chloroform and benzene *via* NMR spectroscopy. The new compounds show a broadening in their absorption bands with absorption above 450 nm, which makes photobleaching with blue light (470 nm) quite efficient. With **4e** a tailing up to 490 nm was observed. All compounds show high quantum yields above 0.5, the only exception is compound **4a**. DSC-measurements show good photopolymerization behavior for all synthesized compounds with double bond conversions comparable to or higher than Ivocerin®. Therefore, these new derivatives can be implemented in a broad field of applications. Further studies to probe the scope of these initiators are currently in progress.

## Author contributions

S. D. P. performed the synthesis towards the diacylgermanes. P. F. performed the Steady-State Photolysis and Determination of Quantum Yields. S. M. M. did the Photo-DSC measurements. S. H. W. and A-M. K. performed the DFT computations and analysed the results. A. T. and R. C. F. measured the X-ray structures. S. D. P., S. M. M. and P. F. jointly wrote the manuscript with help from T. G., G. G. and M. H. All authors discussed the results and commented on the manuscript. M. H. provided supervision and wrote the final version of the manuscript.

## Conflicts of interest

There are no conflicts to declare.

## Supplementary Material

DT-050-D1DT02091A-s001

DT-050-D1DT02091A-s002

DT-050-D1DT02091A-s003
