# Correction: Synthesis and characterization of diacylgermanes: persistent derivatives with superior photoreactivity

**DOI:** 10.1039/d2dt90022j

**Published:** 2022-02-07

**Authors:** Sabrina D. Püschmann, Philipp Frühwirt, Stefanie M. Müller, Stefan H. Wagner, Ana Torvisco, Roland C. Fischer, Anne-Marie Kelterer, Thomas Griesser, Georg Gescheidt, Michael Haas

**Affiliations:** Institute of Inorganic Chemistry, Technical University Graz Stremayrgasse 9/IV 8010 Graz Austria michael.haas@tugraz.at; Institute of Physical and Theoretical Chemistry, Technical University Graz Stremayrgasse 9/II 8010 Graz Austria; Institute of Chemistry of Polymeric Materials, Montanuniversitaet Leoben Otto-Gloeckelstrasse 2 A-8700 Leoben Austria

## Abstract

Correction for ‘Synthesis and characterization of diacylgermanes: persistent derivatives with superior photoreactivity’ by Sabrina D. Püschmann *et al.*, *Dalton Trans.*, 2021, **50**, 11965–11974, DOI: 10.1039/D1DT02091A.

The authors regret that the HRMS data in the original manuscript were erroneously reported as being acquired in FD^+^ mode rather than the correct negative LIFDI mode. This led to some of the HRMS data being incorrect for compounds reported in the manuscript. The correct values for the affected compounds are listed below:

Dimesityldimesitoylgermane (**4a**) HRMS: (LIFDI^−^) calcd for [C_38_H_44_GeO_2_]^−^ (M^−^): 606.25586. Found: 606.3292.

Dimesityldibenzoylgermane (**4b**) HRMS: (LIFDI^−^) calcd for [C_32_H_32_GeO_2_]^−^ (M^−^): 522.16196. Found: 522.2216.

Dimesityldi(*o*-toluoyl)germane (**4c**) HRMS: (LIFDI^−^) calcd for [C_34_H_36_GeO_2_]^−^ (M^−^): 550.19326. Found: 550.2034.

Dimesityldibenzothiophenegermane (**4e**) HRMS: (LIFDI^−^) calcd for [C_36_H_32_O_2_S_2_Ge]^−^ (M^−^): 634.10555. Found: 634.1927.

 

The Royal Society of Chemistry apologises for these errors and any consequent inconvenience to authors and readers.

## Supplementary Material

