# Supplementary material for: Synthesis and characterization of diacylgermanes: persistent derivatives with superior photoreactivity
Source: Dalton Trans. 2021 Aug 5;50(34):11965–74. doi: 10.1039/d1dt02091a (PMC8406493; doi:10.1039/d1dt02091a)
Supplement: DT-050-D1DT02091A-s002 [file DT-050-D1DT02091A-s002.pdf]

Electronic Supplementary Material (ESI) for Dalton Transactions.
This journal is © The Royal Society of Chemistry 2021

Supporting Information

Synthesis and characterization of diacylgermanes: persistent derivatives with superior photoreactivity

Sabrina D. Püschmann, [a] *Philipp Frühwirt,* [b] *Stefanie M. Müller,*[c] *Stefan H. Wagner,* [b]*Ana Torvisco,* [a] *Roland C. Fischer,* [a] *Anne-Marie Kelterer,* [b] *Thomas Griesser,*[c] *Georg Gescheidt,* [b] *Michael Haas** [a]

[a] Institute of Inorganic Chemistry, Technical University Graz, Stremayrgasse 9/IV, 8010 Graz (Austria).

[b] Institute of Physical and Theoretical Chemistry, Technical University Graz, Stremayrgasse 9/II, 8010 Graz (Austria).

[c] Institute of Chemistry of Polymeric Materials, Montanuniversitaet Leoben, Otto-Gloeckelstrasse 2, A-8700 Leoben (Austria).

Table of Contents

1. NMR Spectroscopy 3
  1.1. Dichlorodimesitylgermane **(1)** 3
  1.2. Dimesityldi(trimethylsilyl)germane **(2)** 4
  1.3. Germanide **(3)** 4
  1.4. Dimesityldimesitoylgermane **(4a)** 5
  1.5. Dimesityldibenzoylgermane **(4b)** 6
  1.6. Dimesityldi(*o*-toluoyl)germane **(4c)** 7
  1.7. Dimesityldi(*o*-methoxy)germane **(4d)** 8
  1.8. Dimesityldibenzothiophenegermane **(4e)** 9
  1.9. Dimesityldibenzofurangermane **(4f)** 10
2. X-ray Crystallography 11
3. Long-Term Storage Stability 14
  3.1. *Via* NMR Spectroscopy 14
    3.1.1. Dimesityldimesitoylgermane **(4a)** 14
    3.1.2. Dimesityldibenzoylgermane **(4b)** 15
    3.1.3. Dimesityldi(*o*-toluoyl)germane **(4c)** 16
    3.1.4. Dimesityldi(*o*-methoxy)germane **(4d)** 17
  3.2. *Via* UV-Vis Spectroscopy 19
4. Steady-State Photolysis and Determination of Quantum Yields 21
5. Photo-DSC measurements 25
6. DFT Calculations 26
7. References 29

1. NMR Spectroscopy

1.1. Dichlorodimesitylgermane **(1)**

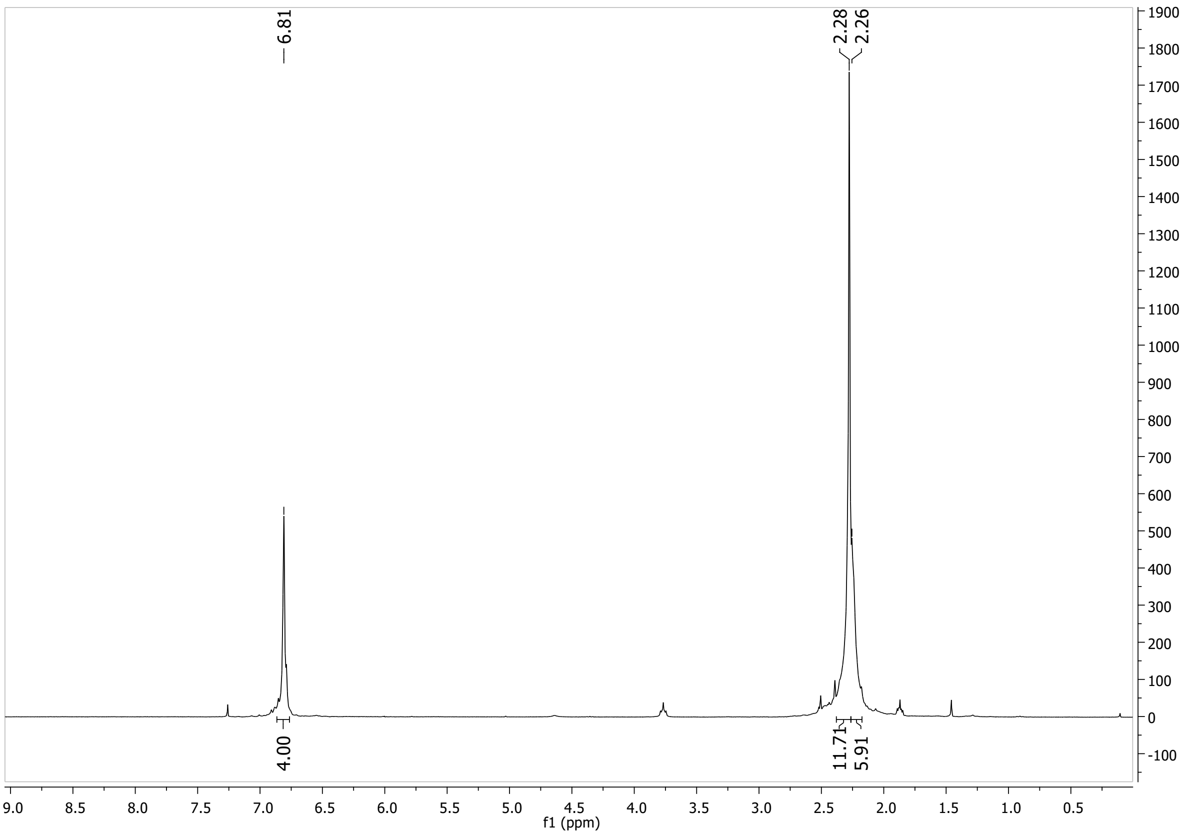

Figure S1. ^{1}H NMR Spectrum (300 MHz, $CDCl_3$) of Dichlorodimesitylgermane **(1)**

1.2. Dimesityldi(trimethylsilyl)germane **(2)**

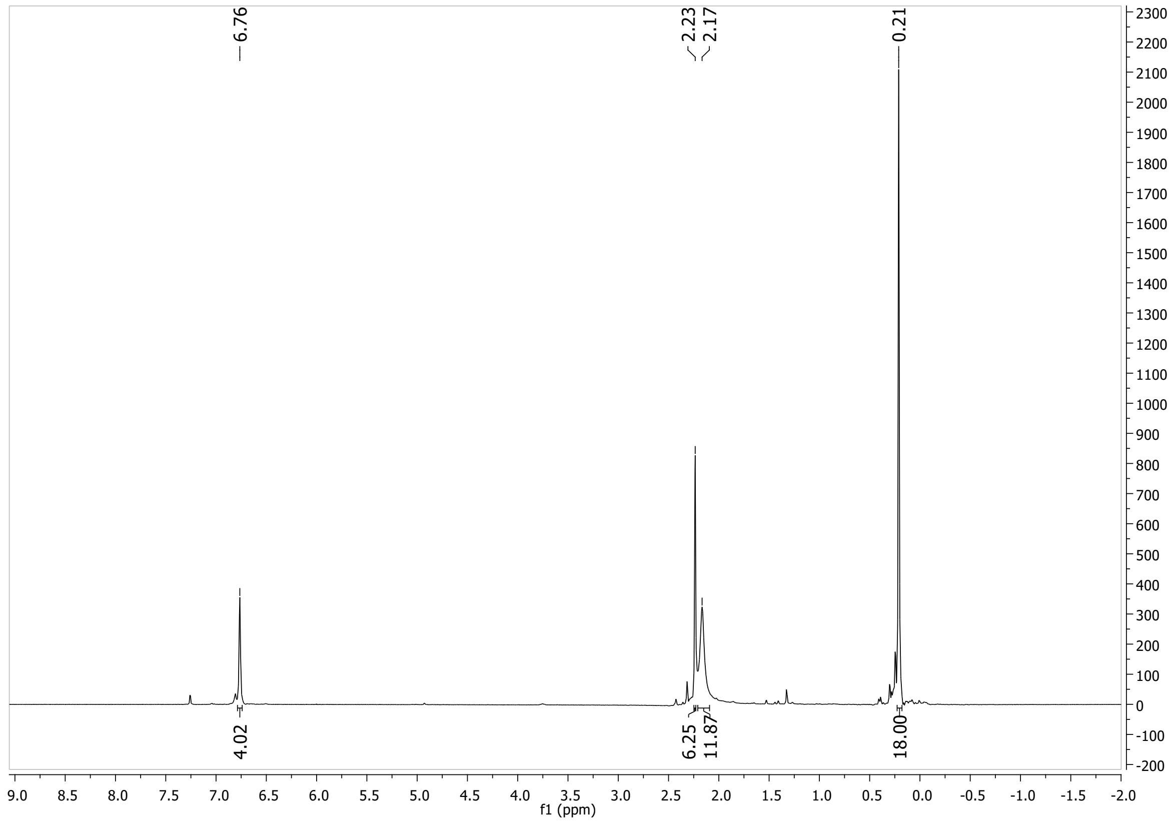

Figure S2. ^{1}H NMR Spectrum (300 MHz, $CDCl_3$) of Dimesityldi(trimethylsilyl)germane **(2)**

1.3. Germanide **(3)**

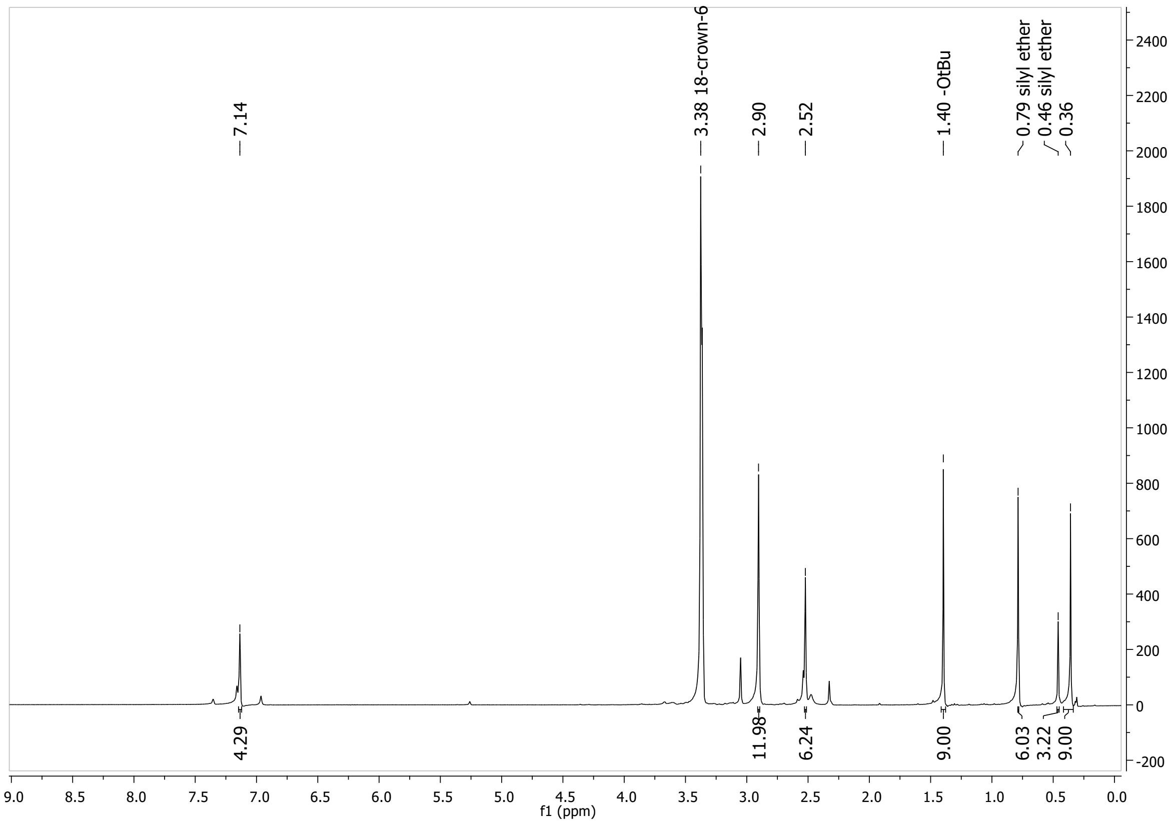

Figure S3. ^{1}H NMR Spectrum (300 MHz, $CDCl_3$) of the intermediate, the germanide **(3)**

1.4. Dimesityldimesitoylgermane **(4a)**

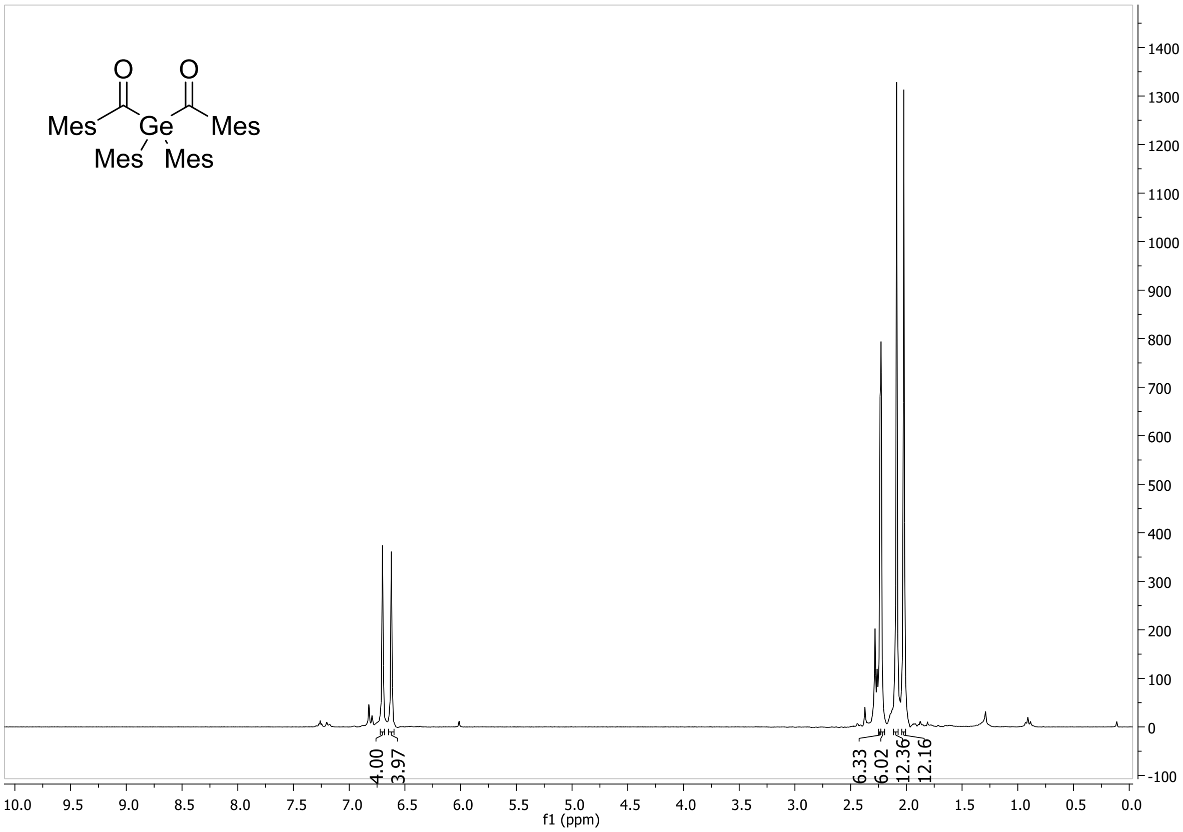

Figure S4. ^{1}H NMR Spectrum (300 MHz, $CDCl_3$) of dimesityldimesitoylgermane **(4a)**

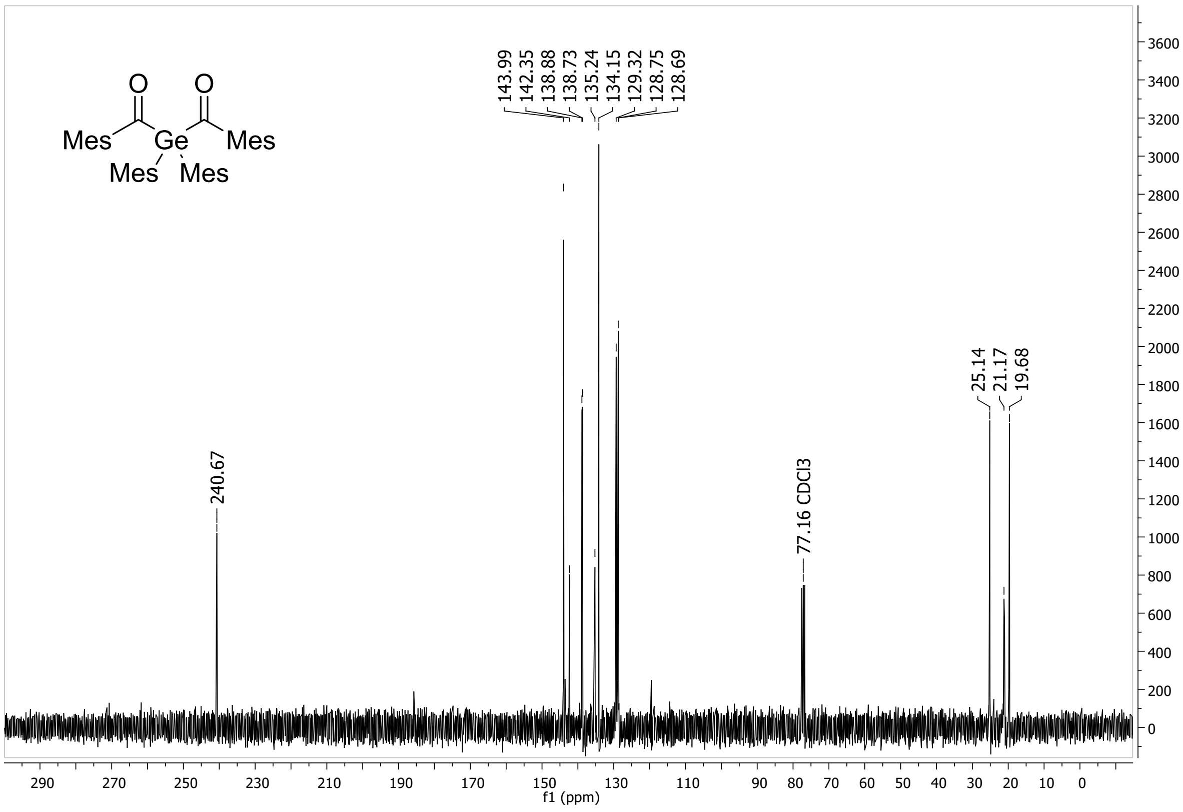

Figure S5. ^{13}C NMR Spectrum (75 MHz, $CDCl_3$) of dimesityldimesitoylgermane **(4a)**

1.5. Dimesityldibenzoylgermane **(4b)**

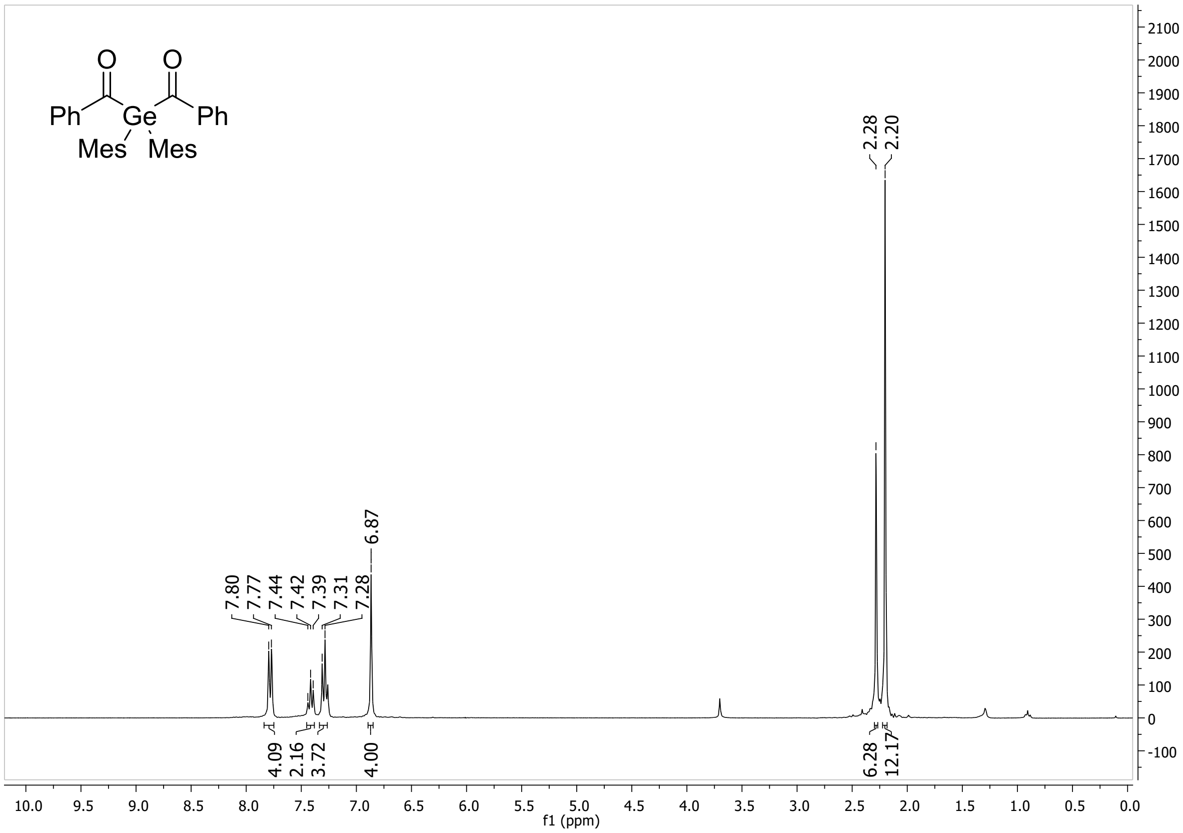

Figure S6. ^{1}H NMR Spectrum (300 MHz, $CDCl_3$) of dimesityldibenzoylgermane **(4b)**

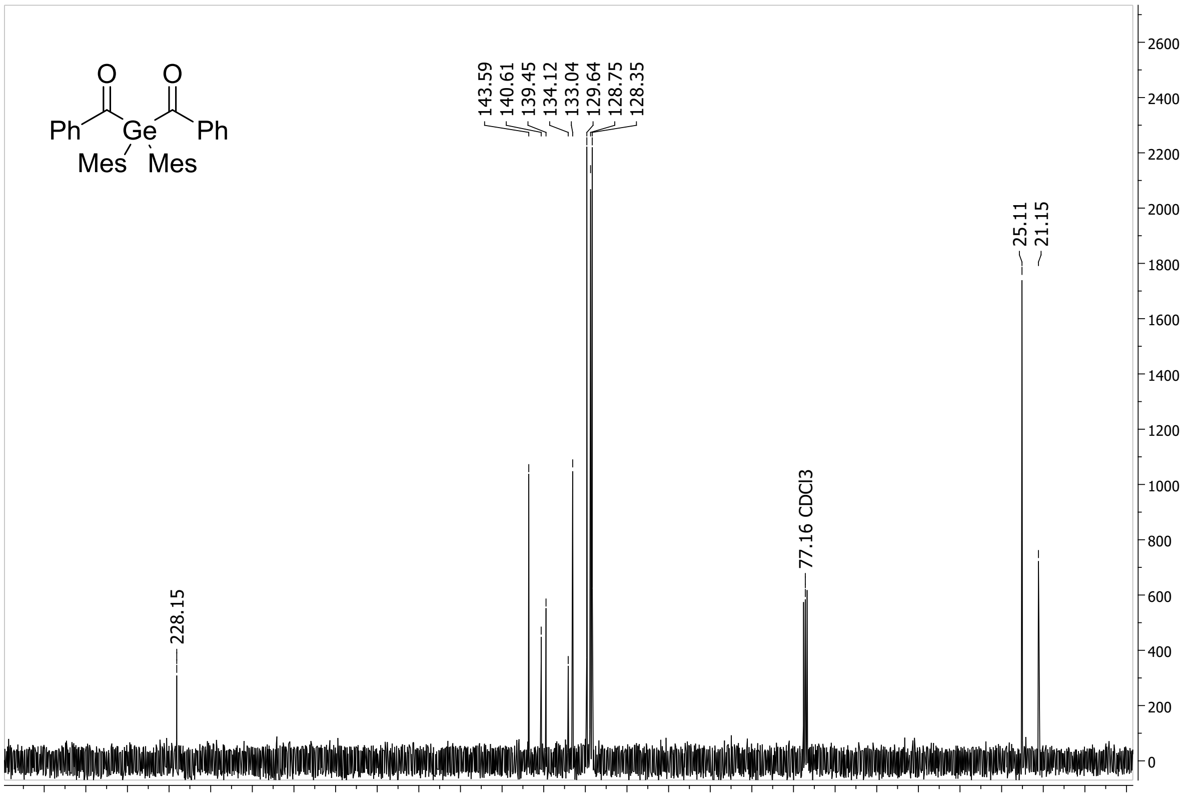

Figure S7. ^{13}C NMR Spectrum (75 MHz, $CDCl_3$) of dimesityldibenzoylgermane **(4b)**

1.6. Dimesityldi(*o*-toluoyl)germane **(4c)**

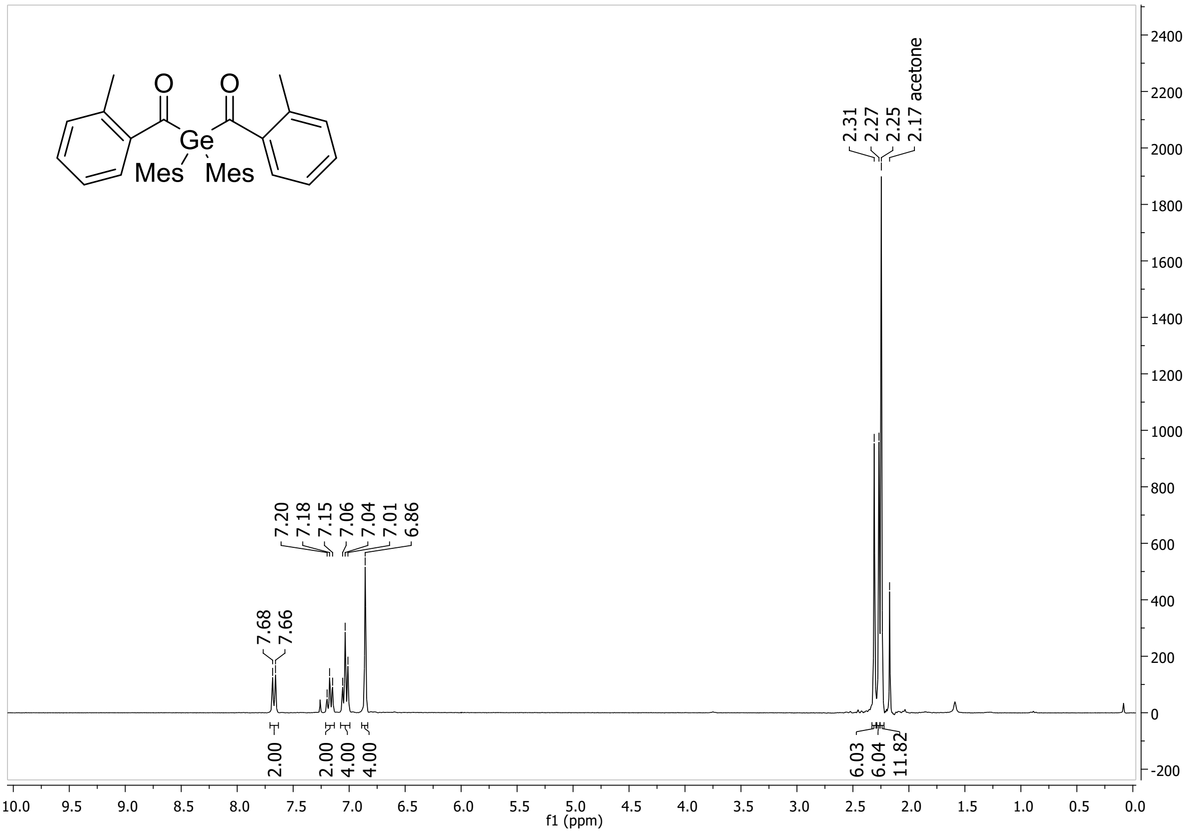

Figure S8. ^{1}H NMR Spectrum (300 MHz, $CDCl_3$) of dimesityldi(*o*-toluoyl)germane **(4c)**

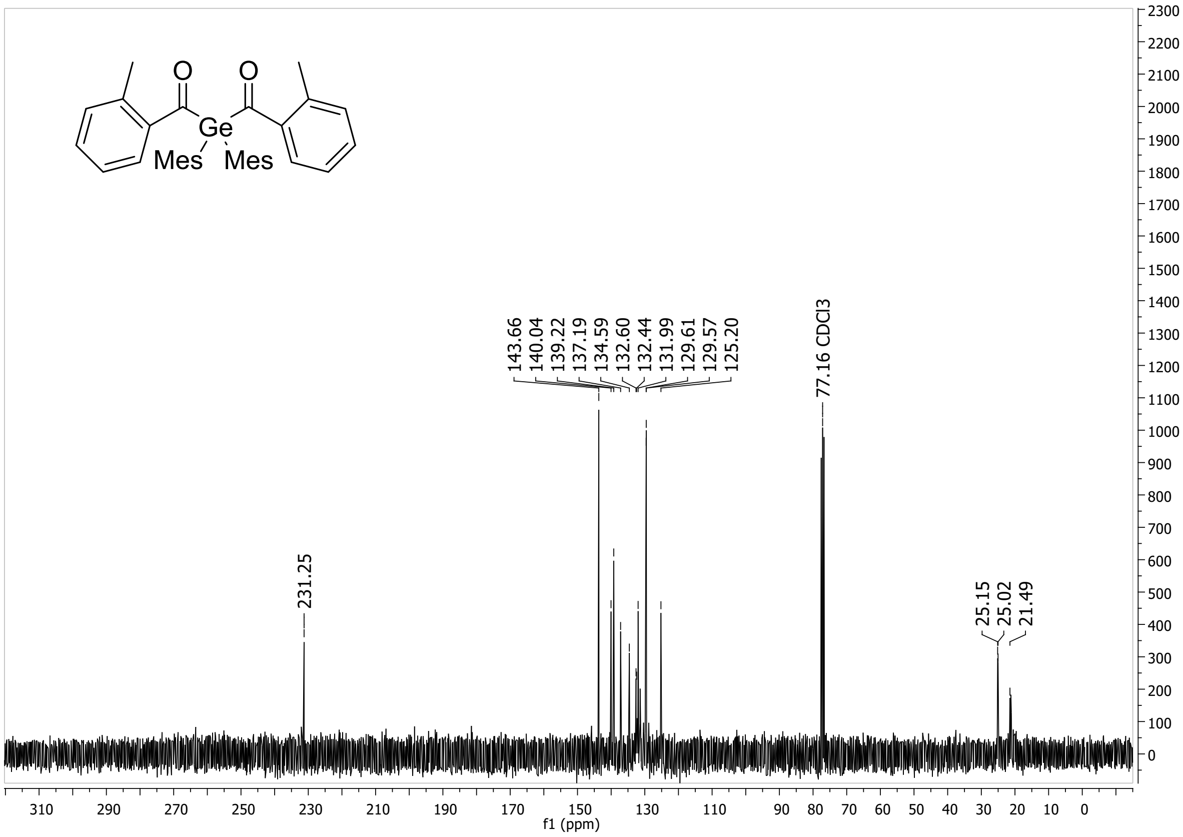

Figure S9. ^{13}C NMR Spectrum (75 MHz, $CDCl_3$) of dimesityldi(*o*-toluoyl)germane **(4c)**

1.7. Dimesityldi(*o*-methoxy)germane **(4d)**

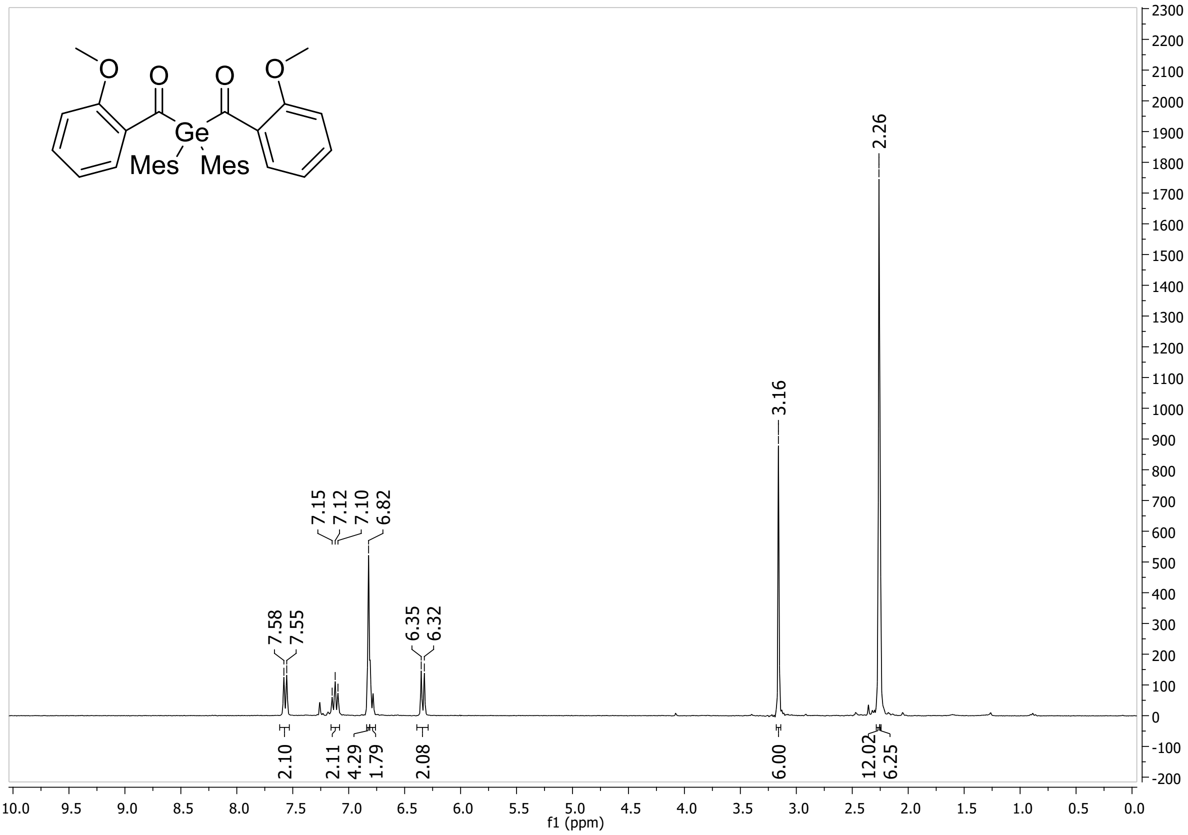

Figure S10. ^{1}H NMR Spectrum (300 MHz, CDCl3) of dimesityldi(*o*-methoxy)germane **(4d)**

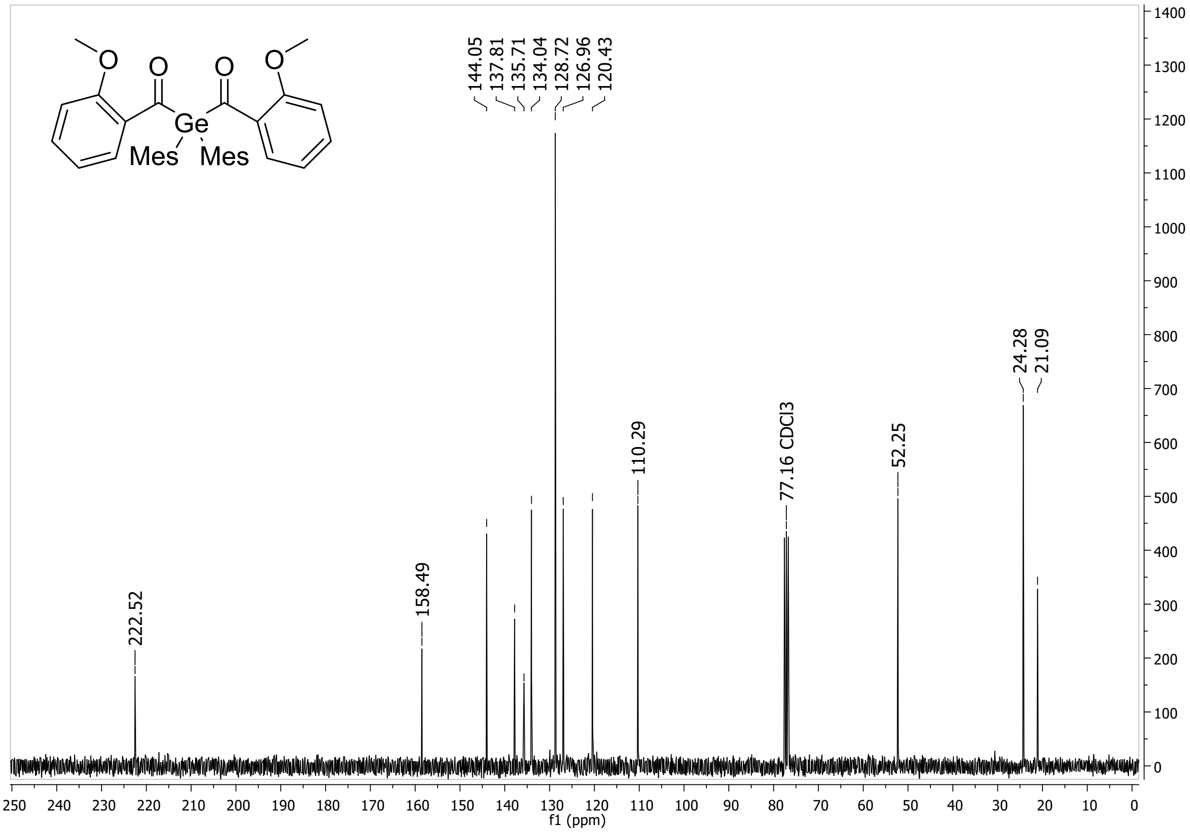

Figure S11. ^{13}C NMR Spectrum (75 MHz, $CDCl_3$) of dimesityldi(*o*-methoxy)germane **(4d)**

1.8. Dimesityldibenzothiophenegermane **(4e)**

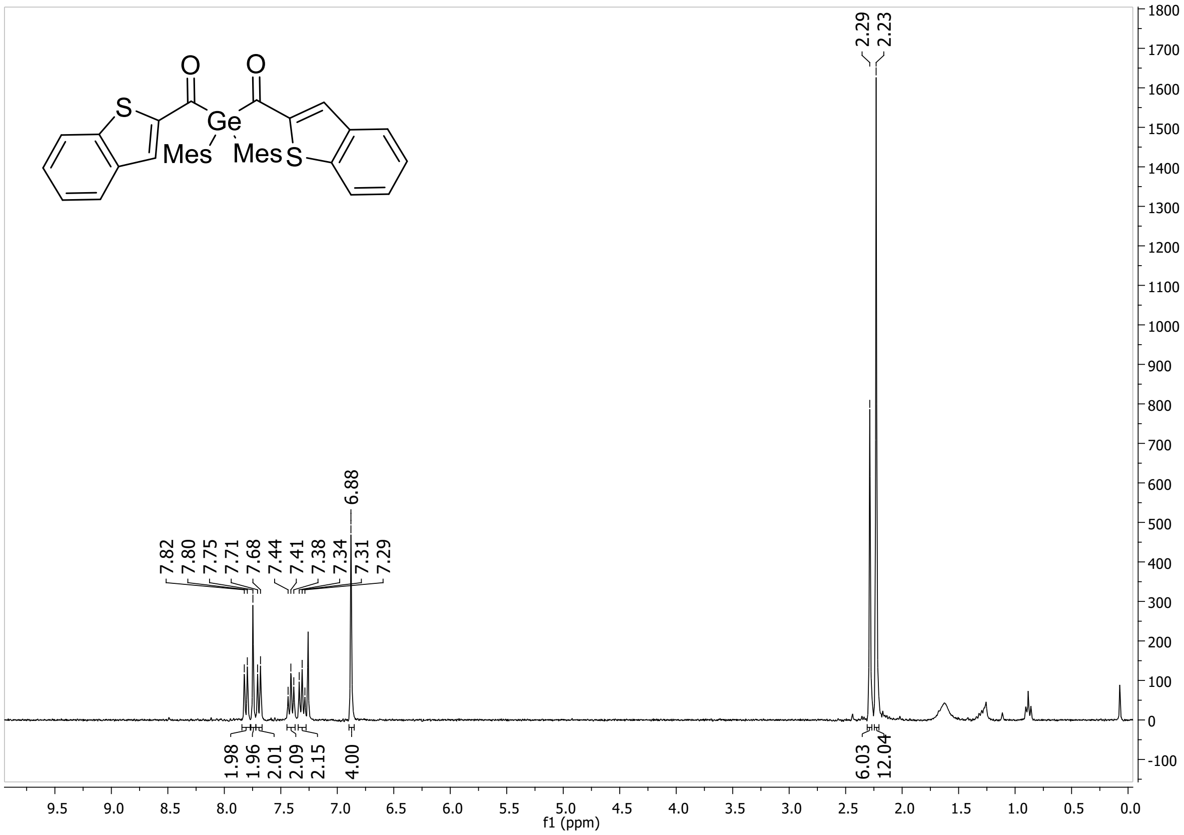

Figure S12. ^{1}H NMR Spectrum (300 MHz, $CDCl_3$) of dimesityldibenzothiophenegermane **(4e)**

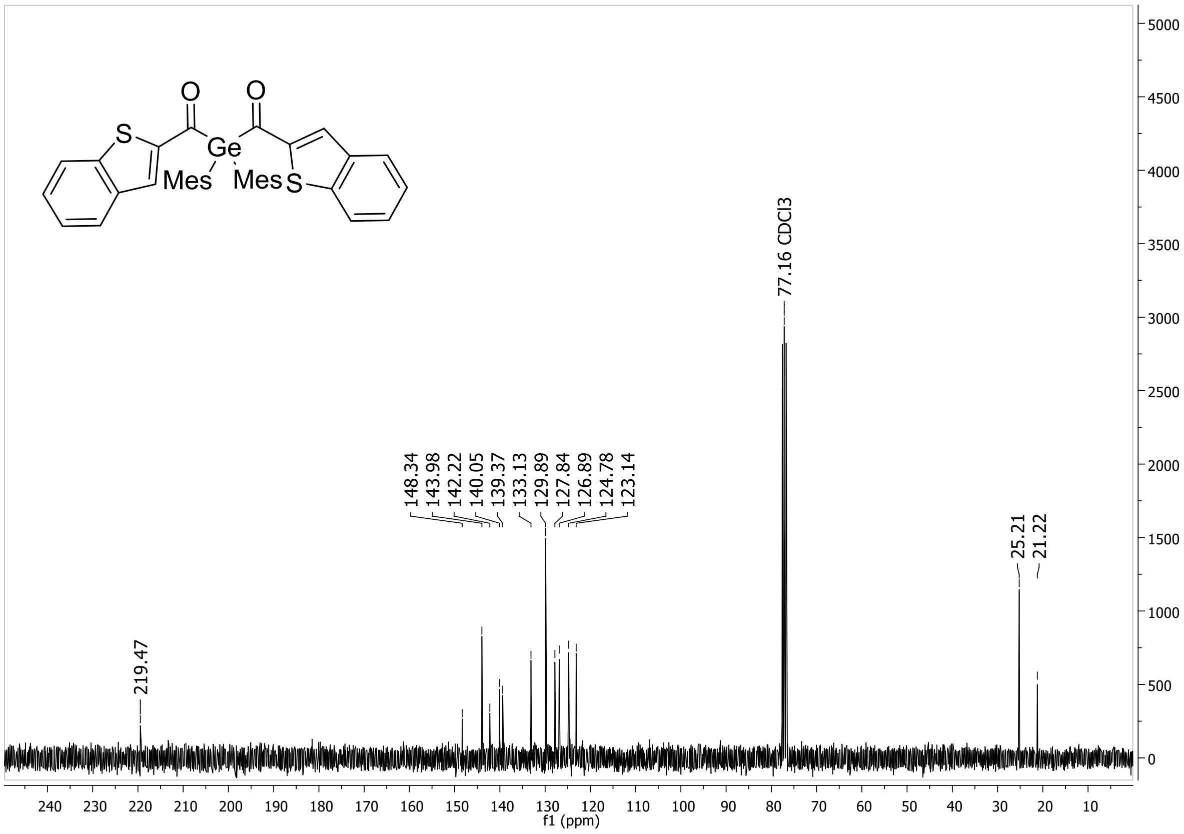

Figure S13. ^{13}C NMR Spectrum (75 MHz, $CDCl_3$) of dimesityldibenzothiophenegermane **(4e)**

1.9. Dimesityldibenzofurangermane **(4f)**

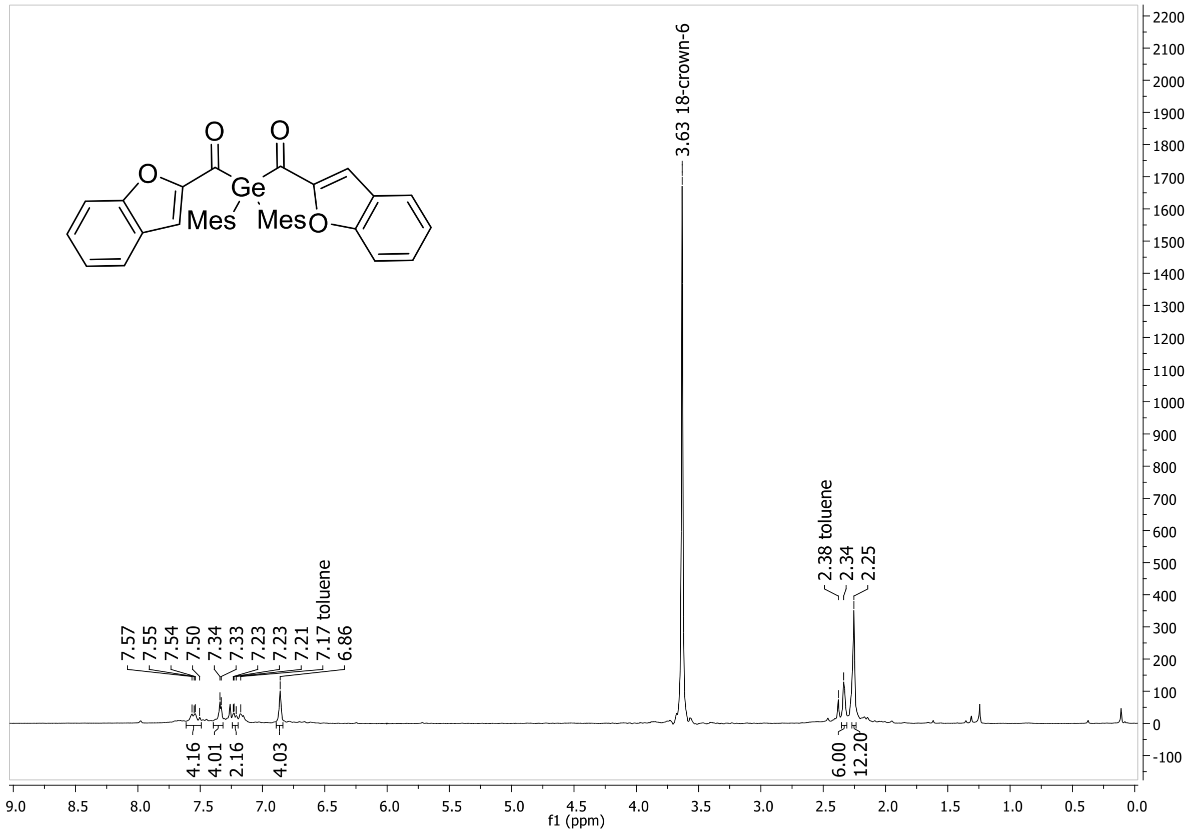

Figure S14. ^{1}H NMR Spectrum (300 MHz, $CDCl_3$) of dimesityldibenzofurangermane **(4f)**

2. X-ray Crystallography

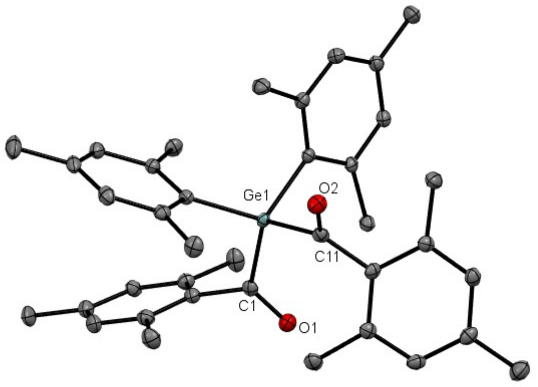

Figure S15. ORTEP representation of **4a**. Thermal ellipsoids are depicted at the 50% probability level. Hydrogen atoms are omitted for clarity. The torsion angle (mean value) between the C=O group and the aromatic ring plane of the Mes group is 68.45°.

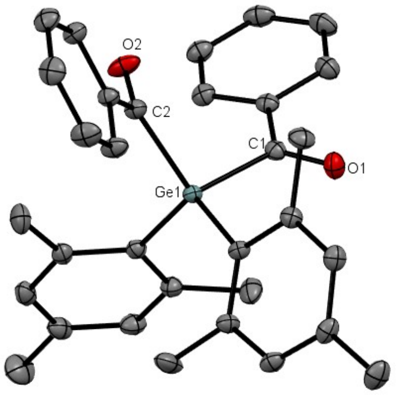

Figure S16. ORTEP representation of **4b**. Thermal ellipsoids are depicted at the 50% probability level. Hydrogen atoms are omitted for clarity. The torsion angle (mean value) between the C=O group and the aromatic ring plane of the benzoyl group is 7.95°.

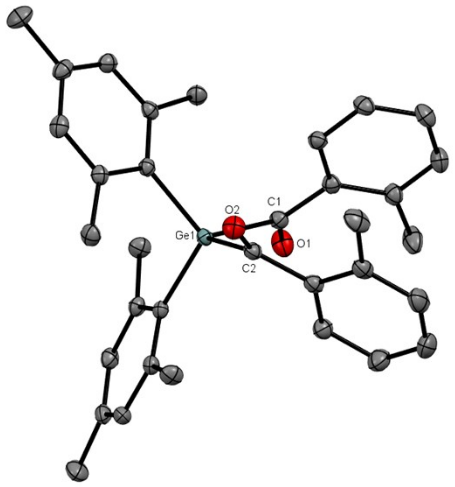

Figure S17. ORTEP representation of **4c**. Thermal ellipsoids are depicted at the 50% probability level. Hydrogen atoms are omitted for clarity. The torsion angle (mean value) between the C=O group and the aromatic ring plane of the *o*-toluoyl group is 25.54°.

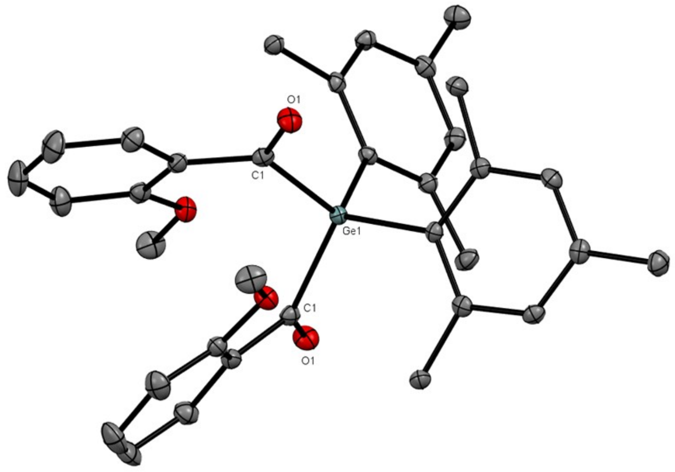

Figure S18. ORTEP representation of **4d**. Thermal ellipsoids are depicted at the 50% probability level. Hydrogen atoms are omitted for clarity. The torsion angle (mean value) between the C=O group and the aromatic ring plane of the *o*-methoxy group is 13.19°.

Table S1. Crystallographic data and details of measurements for compounds **4a**-**e**.

**Compound**	**2078936 - 4a**	**2078938 - 4b**	**2078937 - 4c**	**2078935 - 4d**	**2078939 - 4e**
Formula	$C_{38}H_{44}GeO_2$	$C_{32}H_{32}GeO_2$	$C_{34}H_{36}GeO_2$	$C_{34}H_{36}GeO_4$	$C_{36}H_{32}GeO_2S_2$
Fw (g mol^{-1})	605.32	521.16	549.22	581.24	633.32
a (Å)	13.8778(7)	17.9351(6)	12.7158(4)	16.2702(8)	10.9006(5)
b (Å)	13.9293(7)	9.0656(3)	12.7206(4)	10.6138(4)	28.6448(12)
c (Å)	16.3118(8)	16.5569(5)	18.0599(6)	16.3833(13)	11.1809(5)
α (°)	90	90	90	90	90
β (°)	100.182(3)	101.517(2)	107.229(2)	99.212(5)	118.948(2)
γ (°)	90	90	90	90	90
V ($Å^3$)	3103.5(3)	2637.82(15)	2790.16(16)	2792.7(3)	3055.0(2)
Z	4	4	4	4	4
Crystal size (mm)	0.39 × 0.27 × 0.19	0.20 × 0.19 × 0.16	0.15 × 0.13 × 0.12	0.39 × 0.26 × 0.19	0.39 × 0.15 × 0.09
Crystal habit	Block, yellow	Block, yellow	Block, orange	Block, yellow	Block, yellow
Crystal system	Monoclinic	Monoclinic	Monoclinic	Monoclinic	Monoclinic
Space group	$P2_1/n$	$P2_1/c$	$P2_1/c$	$I2/a$	$P2_1$
d_{calc} (Mg m^{-3})	1.295	1.312	1.307	1.382	1.377
μ (mm^{-1})	1.02	1.19	1.13	1.14	1.17
T (K)	100(2)	100(2)	100(2)	100(2)	100(2)
2*θ* range (°)	2.3–29.7	2.5–32.1	2.3–28.9	2.3–30.3	2.2–28.8
F(000)	1280	1088	1152	1216	1312
T_{min}, T_{max}	0.551, 0.746	0.444, 0.747	0.513, 0.747	0.533, 0.747	0.572, 0.747
R_{int}	0.076	0.110	0.120	0.095	0.110
No. of measured, independent and observed [*I* > 2s(*I*)] reflections	116649, 9063, 7410	137154, 10045, 8053	192347, 10673, 7458	71564, 2735, 2567	333171, 23420, 20378
independent reflections	9063	10045	10673	2735	23420
No. of parameters, restraints	382, 0	322, 0	342, 0	181,0	752, 1
Δ›$_{max}$, Δ›$_{min}$ (e $Å^{-3}$)	1.10, -0.45	0.83, -0.72	0.88, -0.57	0.38, -0.49	0.47, -0.80
R1, wR2 (all data)	R1 = 0.0501 wR2 = 0.0926	R1 = 0.0488 wR2 = 0.0898	R1 = 0.0731 wR2 = 0.1042	R1 = 0.0488 wR2 = 0.0898	R1 = 0.0468 wR2 = 0.0783
R1, wR2 (>2*σ*)	R1 = 0.0353 wR2 = 0.0851	R1 = 0.0335 wR2 = 0.0823	R1 = 0.0391 wR2 = 0.0873	R1 = 0.0335 wR2 = 0.0823	R1 = 0.0348 wR2 = 0.0730

3. Long-Term Storage Stability

3.1. *Via* NMR Spectroscopy

3.1.1. Dimesityldimesitoylgermane **(4a)**

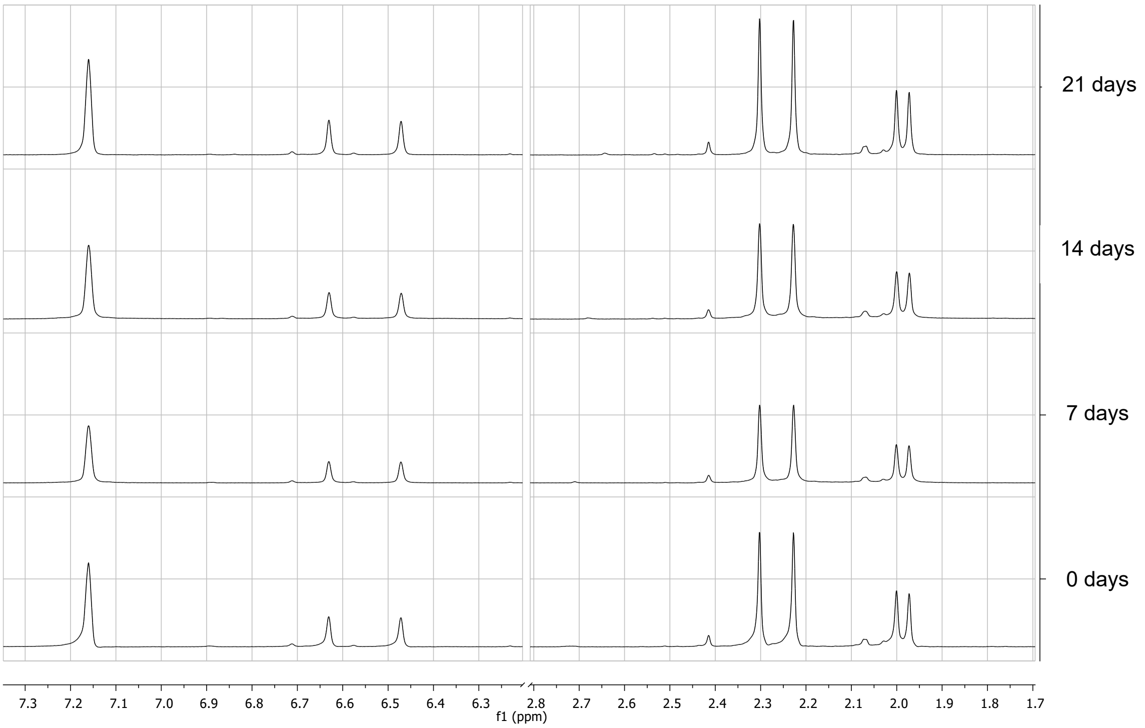

Figure S19. Stacked ^1H NMR Spectrum (300 MHz) of 0.01 M dimesityldimesitoylgermane **(4a)** measured in 0.5 mL degassed C_6D_6 (61.7 ppm H_2O) once a week to get an insight over a long-term storage stability.

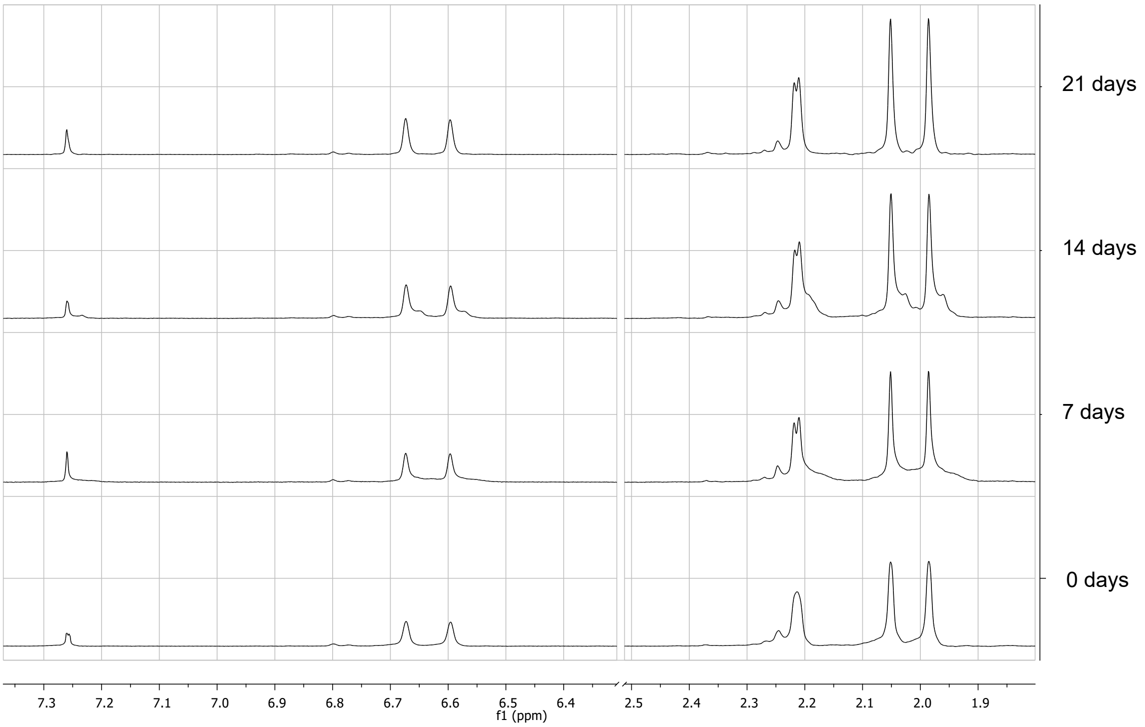

Figure S20. Stacked ^1H NMR Spectrum (300 MHz) of 0.01 M dimesityldimesitoylgermane **(4a)** measured in 0.5 mL degassed $CDCl_3$ (26 ppm H_2O) once a week to get an insight over a long-term storage stability.

3.1.2. Dimesityldibenzoylgermane **(4b)**

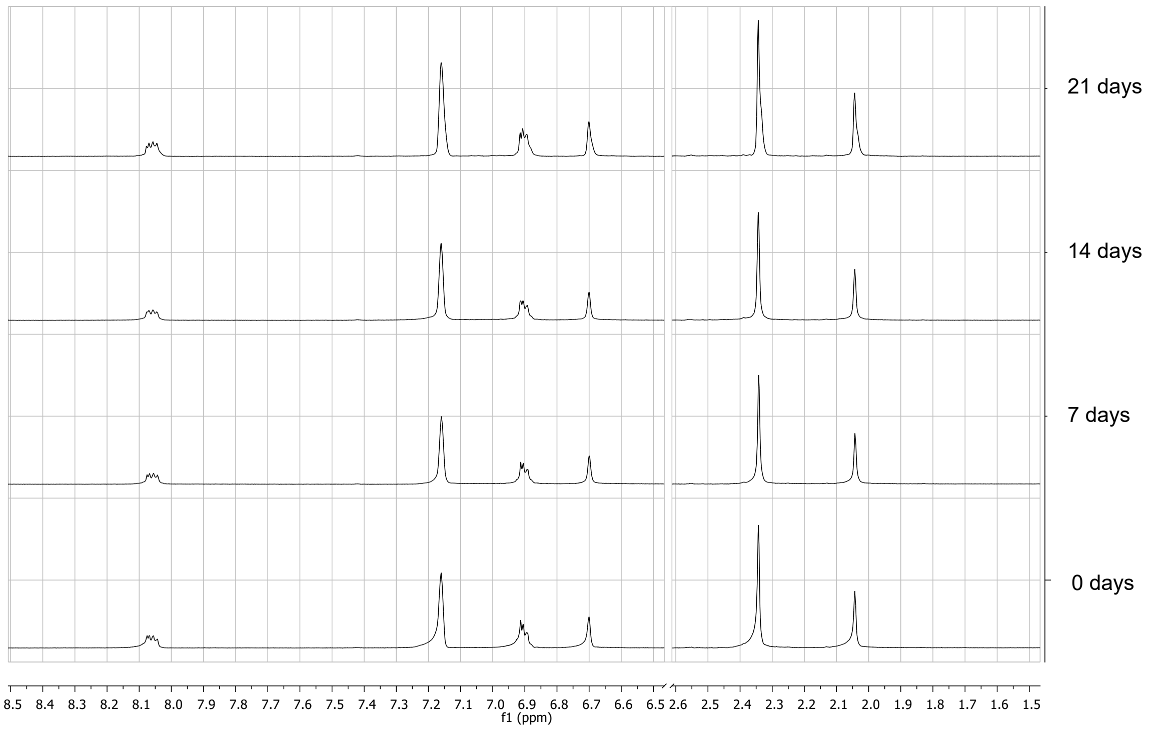

Figure S21. Stacked ^1H NMR Spectrum (300 MHz) of 0.01 M dimesityldibenzoylgermane **(4b)** measured in 0.5 mL degassed C_6D_6 (61.7 ppm H_2O) once a week to get an insight over a long-term storage stability.

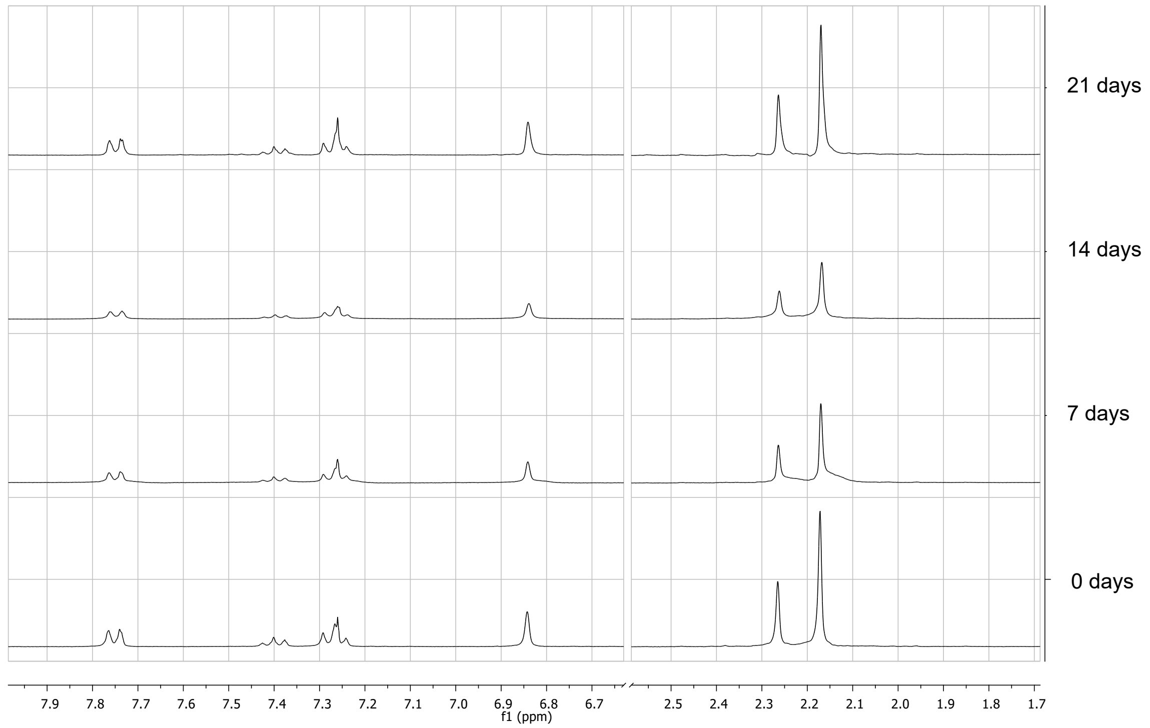

Figure S22. Stacked ^1H NMR Spectrum (300 MHz) of 0.01 M dimesityldibenzoylgermane **(4b)** measured in 0.5 mL degassed $CDCl_3$ (26 ppm H_2O) once a week to get an insight over a long-term storage stability.

3.1.3. Dimesityldi(*o*-toluoyl)germane **(4c)**

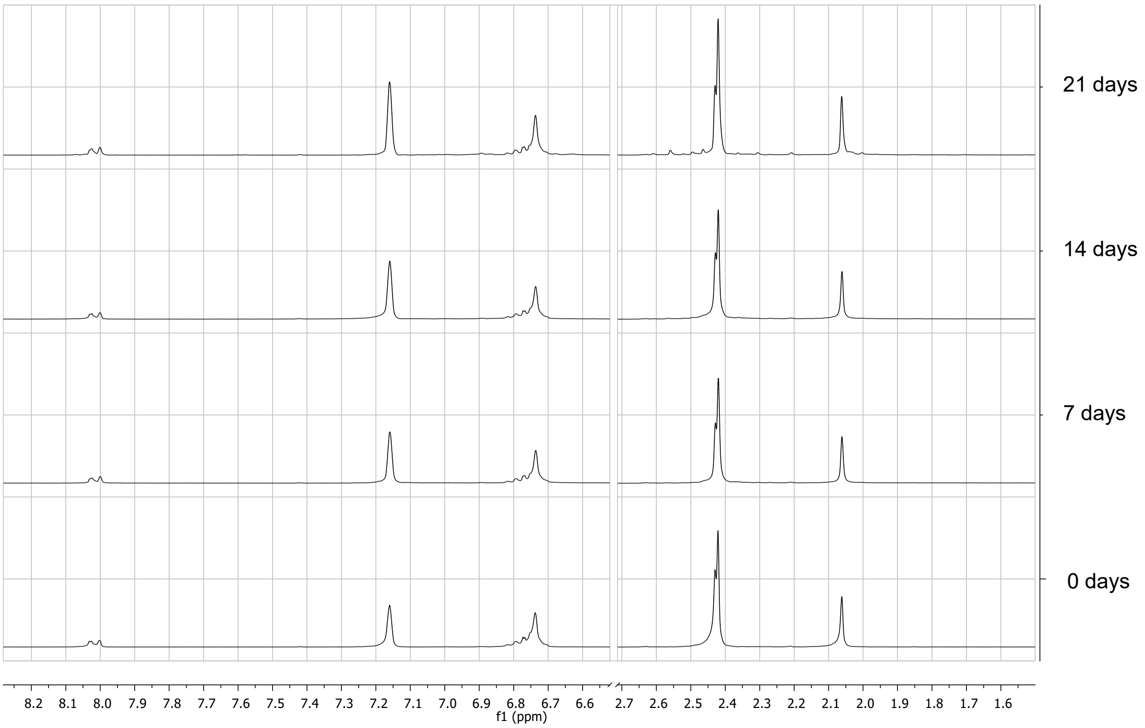

Figure S23. Stacked ^{1}H NMR Spectrum (300 MHz) of 0.01 M dimesityldi(*o*-toluoyl)germane **(4c)** measured in 0.5 mL degassed C_6D_6 (61.7 ppm H_2O) once a week to get an insight over a long-term storage stability.

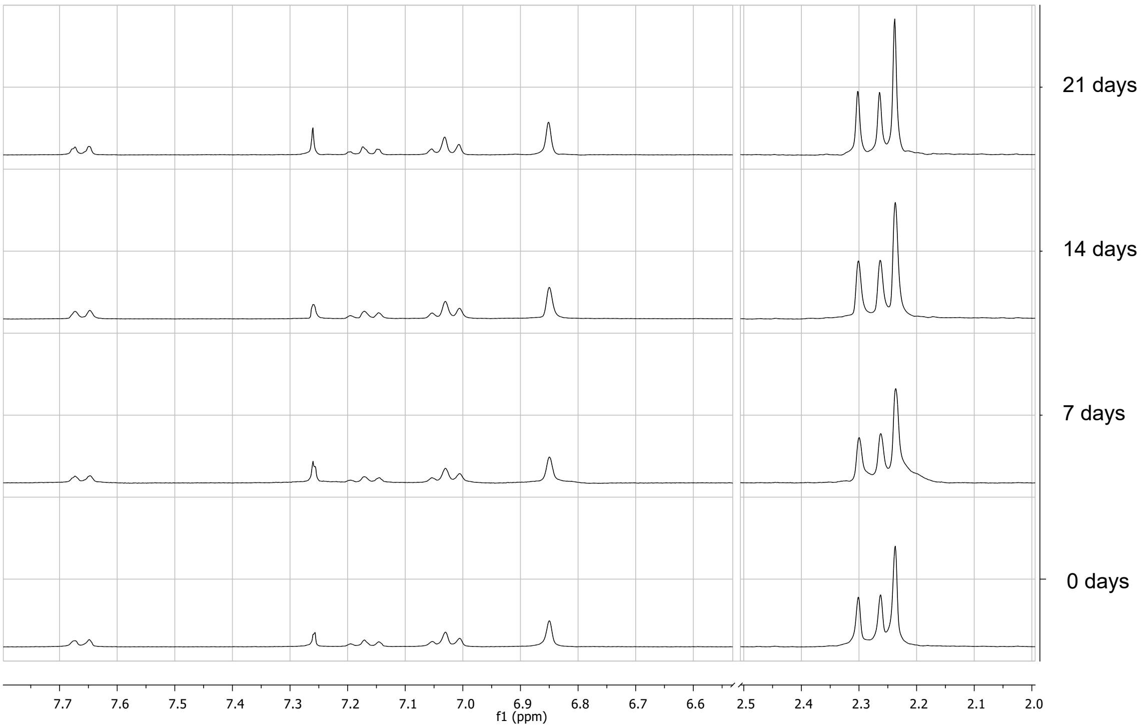

Figure S24. Stacked ^{1}H NMR Spectrum (300 MHz) of 0.01 M dimesityldi(*o*-toluoyl)germane **(4c)** measured in 0.5 mL degassed $CDCl_3$ (26 ppm H_2O) once a week to get an insight over a long-term storage stability.

3.1.4. Dimesityldi(*o*-methoxy)germane **(4d)**

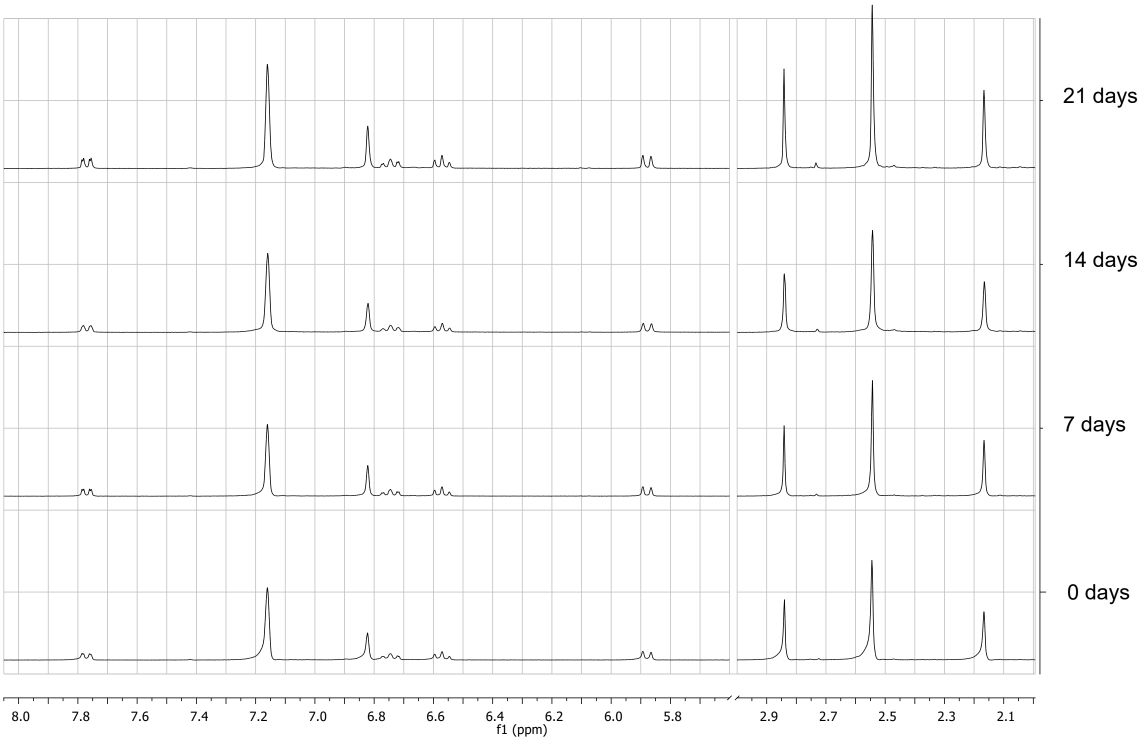

Figure S25. Stacked ^{1}H NMR Spectrum (300 MHz) of 0.01 M dimesityldi(*o*-methoxyl)germane **(4d)** measured in 0.5 mL degassed C_6D_6 (61.7 ppm H_2O) once a week to get an insight over a long-term storage stability.

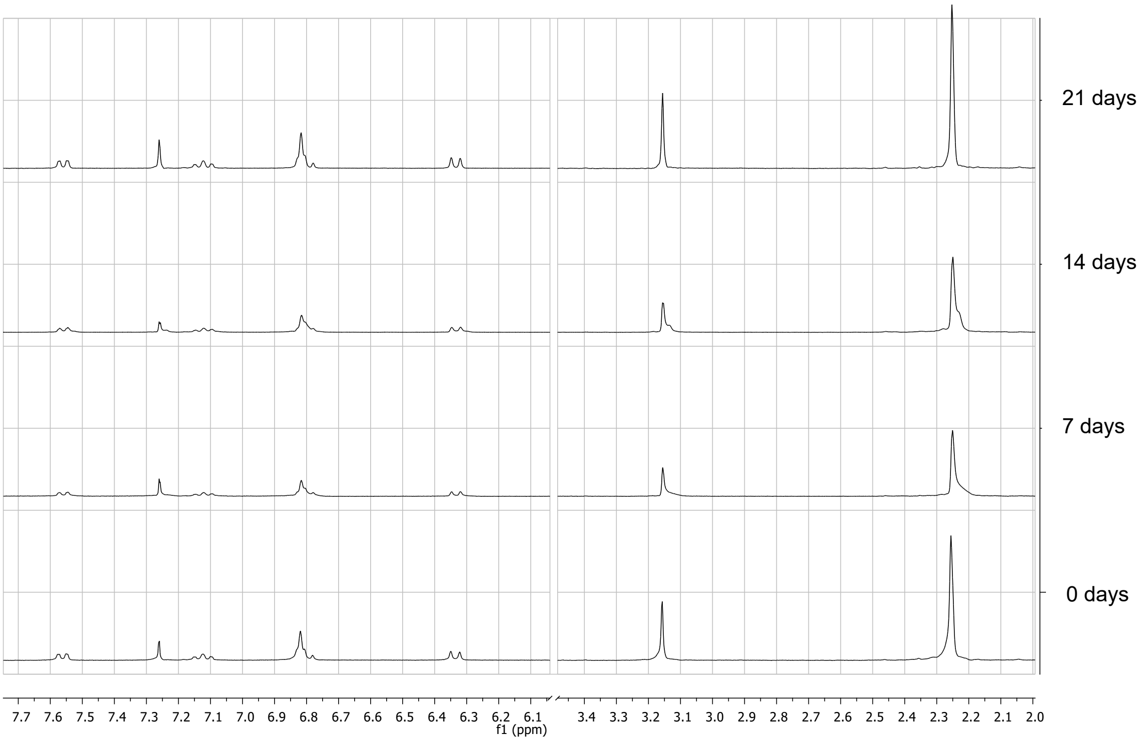

Figure S26. Stacked ^{1}H NMR Spectrum (300 MHz) of 0.01 M dimesityldi(*o*-methoxy)germane **(4d)** measured in 0.5 mL degassed $CDCl_3$ (26 ppm H_2O) once a week to get an insight over a long-term storage stability.

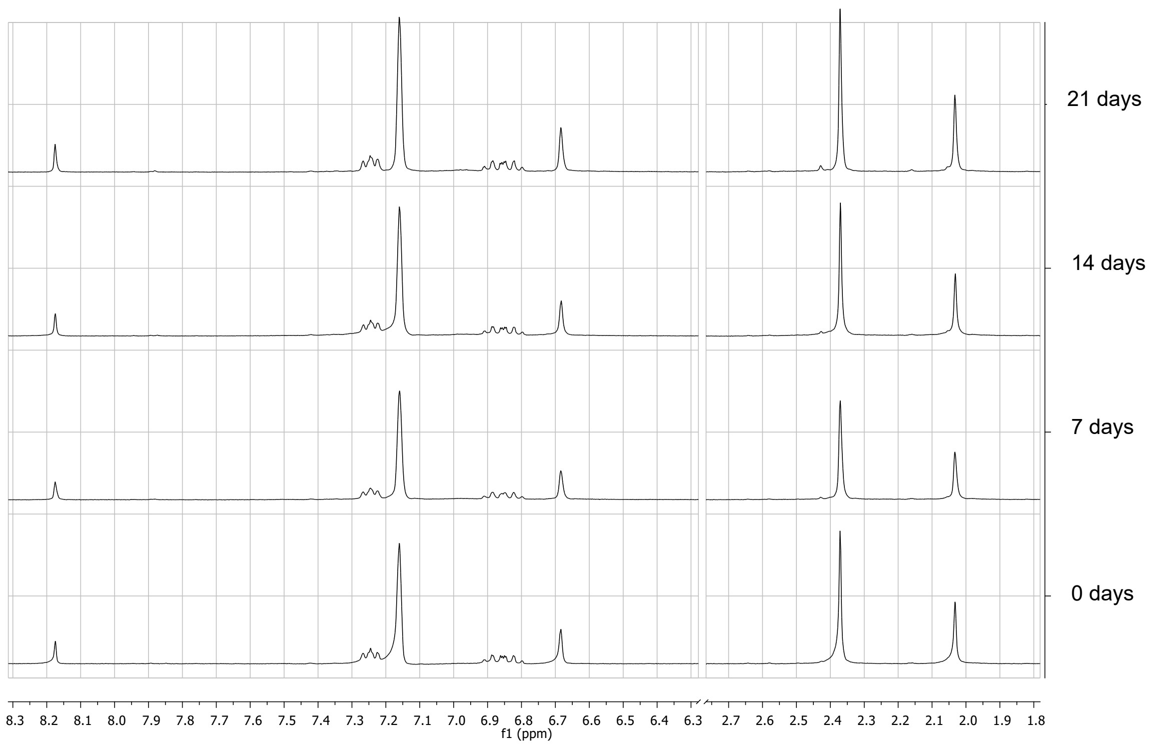

Figure S27. Stacked ^{1}H NMR Spectrum (300 MHz) of 0.01 M dimesityldibenzothiophenegermane **(4e)** measured in 0.5 mL degassed C_6D_6 (61.7 ppm H_2O) once a week to get an insight over a long-term storage stability.

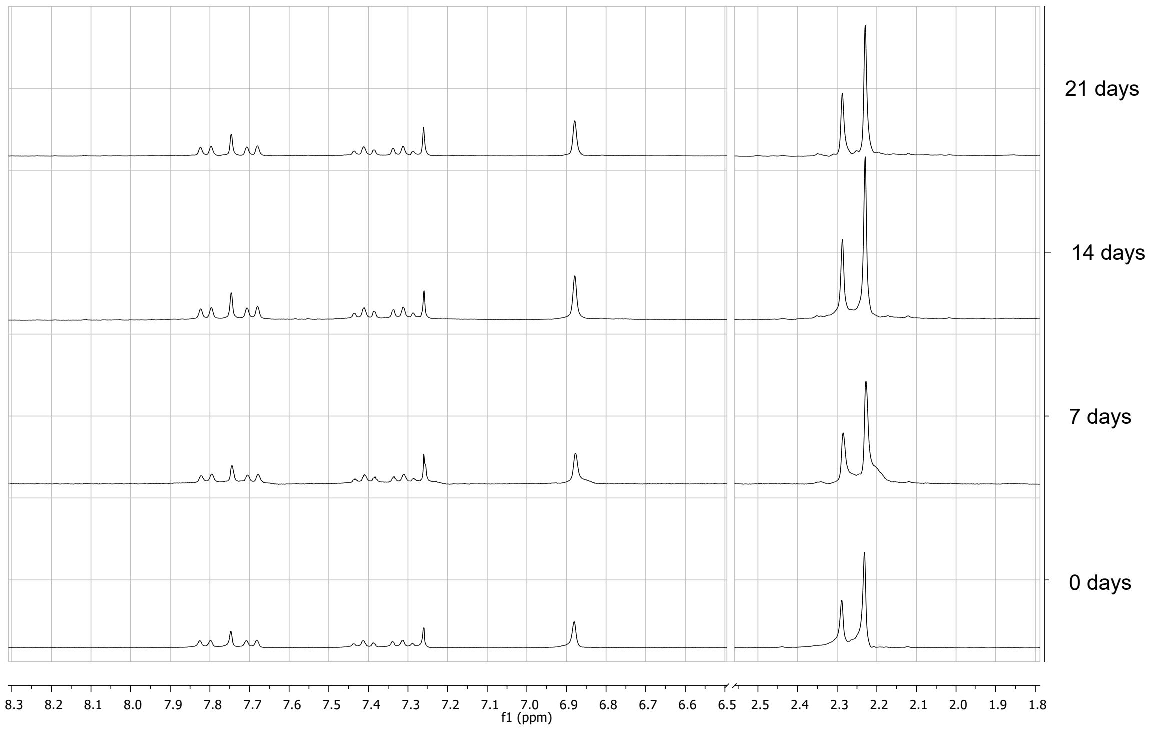

Figure S28. Stacked ^{1}H NMR Spectrum (300 MHz) of 0.01 M dimesityldibenzothiophenegermane **(4e)** measured in 0.5 mL degassed $CDCl_3$ (26 ppm H_2O) once a week to get an insight over a long-term storage stability.

3.2. *Via* UV-Vis Spectroscopy

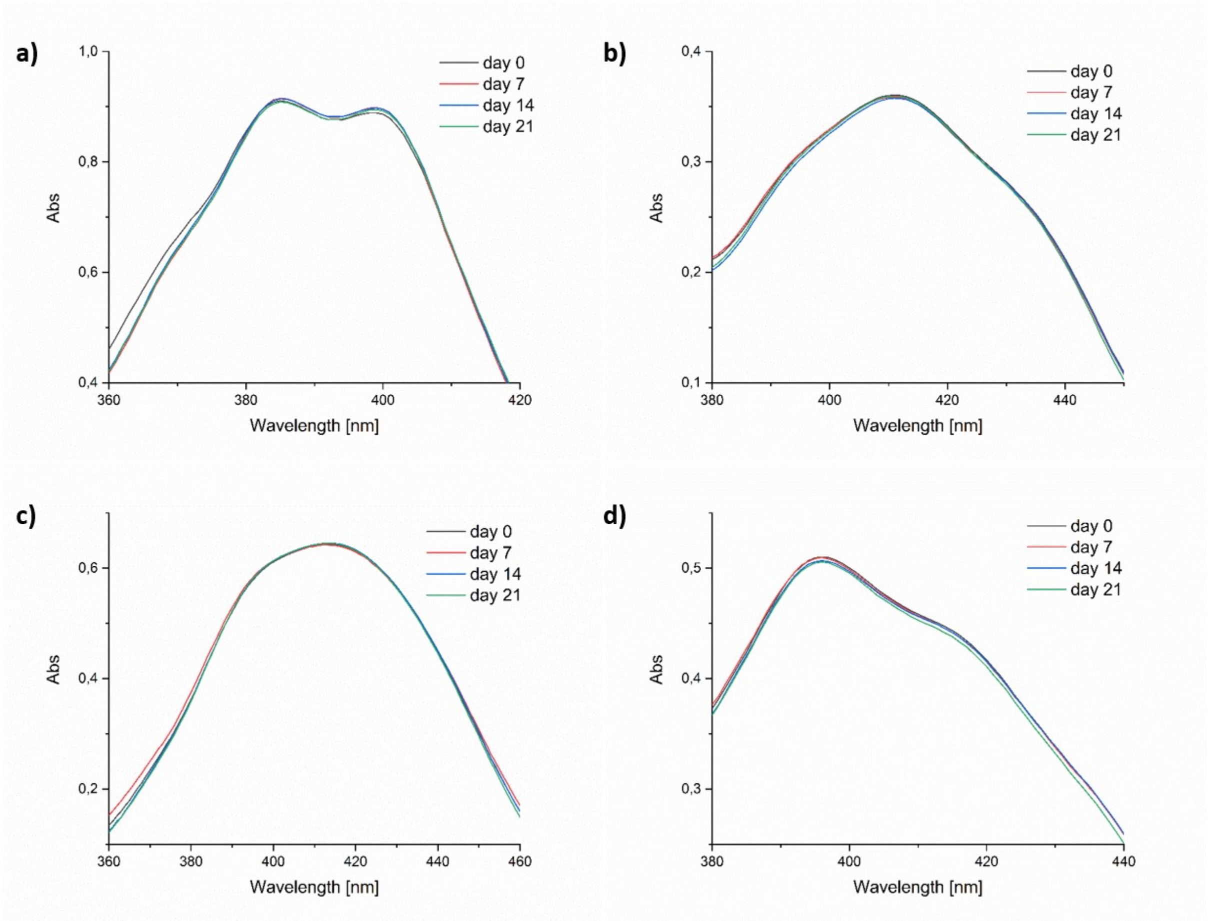

Figure S29. UV-Vis spectra of compounds **4a-d** measured in chloroform with a concentration of 1*10^{-3} M once a week to determine the long-term storage stability of these compounds. **a) 4a**, **b) 4b**, **c) 4c** and **d) 4d**.

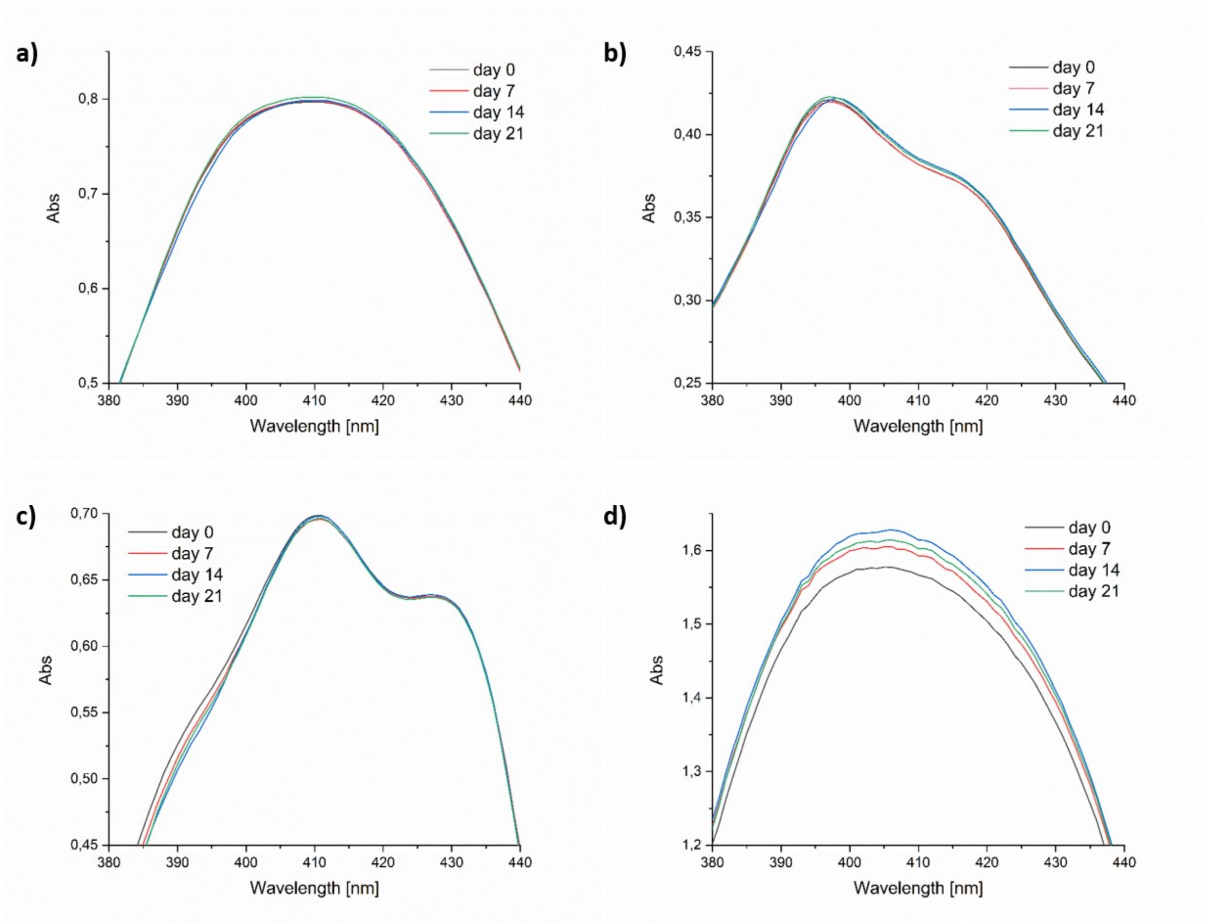

Figure S30. Comparison of the long-term storage stability in MMA with a concentration of $1*10^{-3}$ M detected by UV-Vis spectroscopy over 21 days. **a) 4c**, **b) 4d** and **c) Ivocerin**® and **d) 5**. The absorption of **5** increases due to solvent evaporation over time.

4. Steady-State Photolysis and Determination of Quantum Yields

UV-Vis spectra were acquired on a TIDAS UV-Vis spectrometer equipped with optical fibers and a 1024-pixel diode-array detector (J&M Analytik AG, Essingen, Germany). For the photobleaching experiments, two different LEDs (Roithner Lasertechnik GmbH, Vienna, Austria) – LED385 (emission maximum 387 nm; 15 nm full width at half maximum; 7.8 mW at 20 mA) and LED470 (emission maximum at 463 nm; 30 nm full width at half maximum; 7.4 mW at 20 mA) – were used, which are also found in commercial dental lamps.[3] The output power of the LEDs was determined by a spectrophotometer (GL Spectis, GL Optics, Germany) equipped with an integrating sphere (Ulbricht sphere). Both LEDs were operated at a photon flux of 0.05 µmol s^{-1} to ensure comparability of the results. Solutions of the investigated compounds in a 1:1 (v:v) mixture of toluene and methyl methacrylate (MMA) were filled into 1 cm x 1 cm quartz cuvettes intended for fluorescence measurements and degassed by bubbling with argon for 5 minutes. Samples were irradiated perpendicular to the optical path of the spectrometer and stirred during the measurements with a magnetic stirring bar (750 rpm). The concentrations of the compounds were between 0.76 and 2.01 mM (within the linear range of the spectrometer). The data acquired in the photobleaching experiment with LED385 were used to determine the quantum yields of decomposition. To that end, the absorbance traces were converted to concentration traces and fitted with a mono-exponential function. The setup and method are described in more detail in ref.[4]

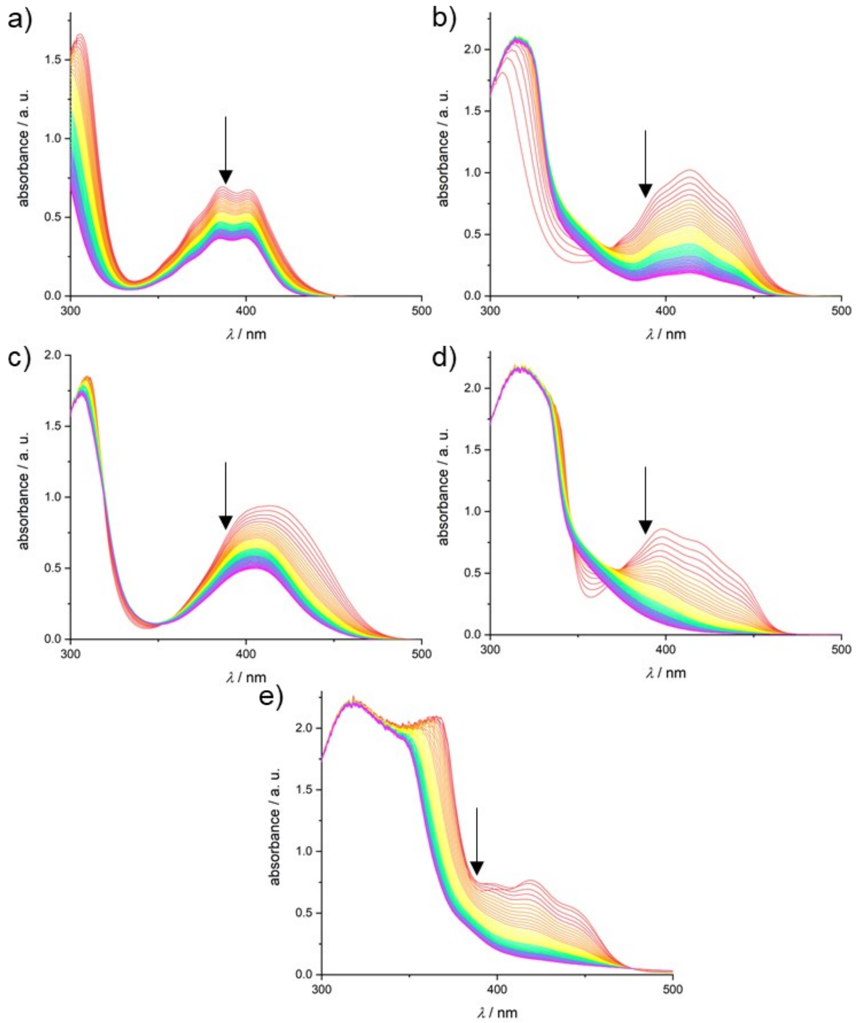

Figure S31. UV-VIS spectra of a) **4a** (0.76 mM), b) **4b** (2.01 mM), c) **4c** (1.51 mM), d) **4d** (1.74 mM), e) **4e** (1.26 mM) in toluene/MMA 1/1 (v/v) during illumination with LED385. Spectra were acquired every 12 seconds. The arrows indicate the emission maximum of the LED.

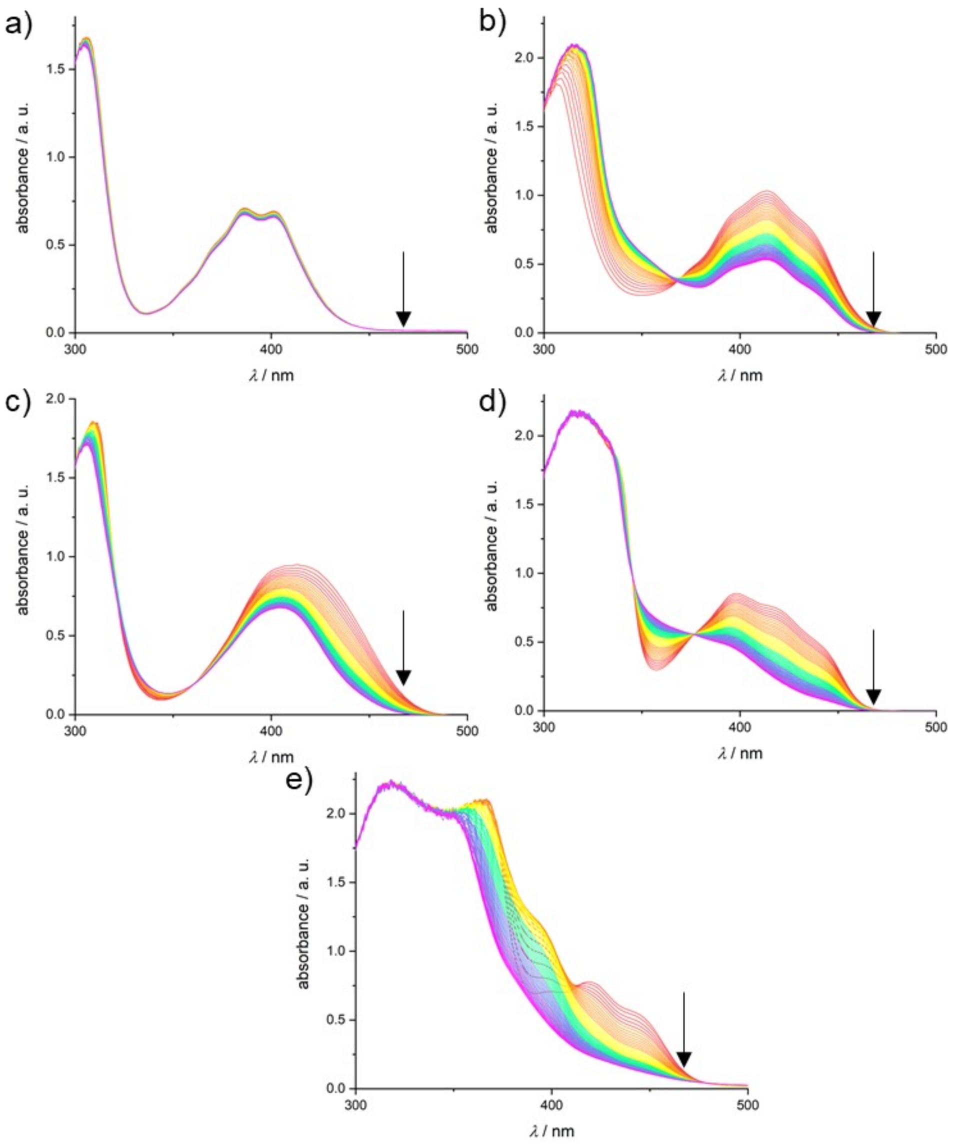

Figure S32. UV-VIS spectra of a) **4a** (0.76 mM), b) **4b** (2.01 mM), c) **4c** (1.51 mM), d) **4d** (1.74 mM), e) **4e** (1.26 mM) in toluene/MMA 1/1 (v/v) during illumination with LED470. Spectra were acquired every 12 seconds. The arrows indicate the emission maximum of the LED.

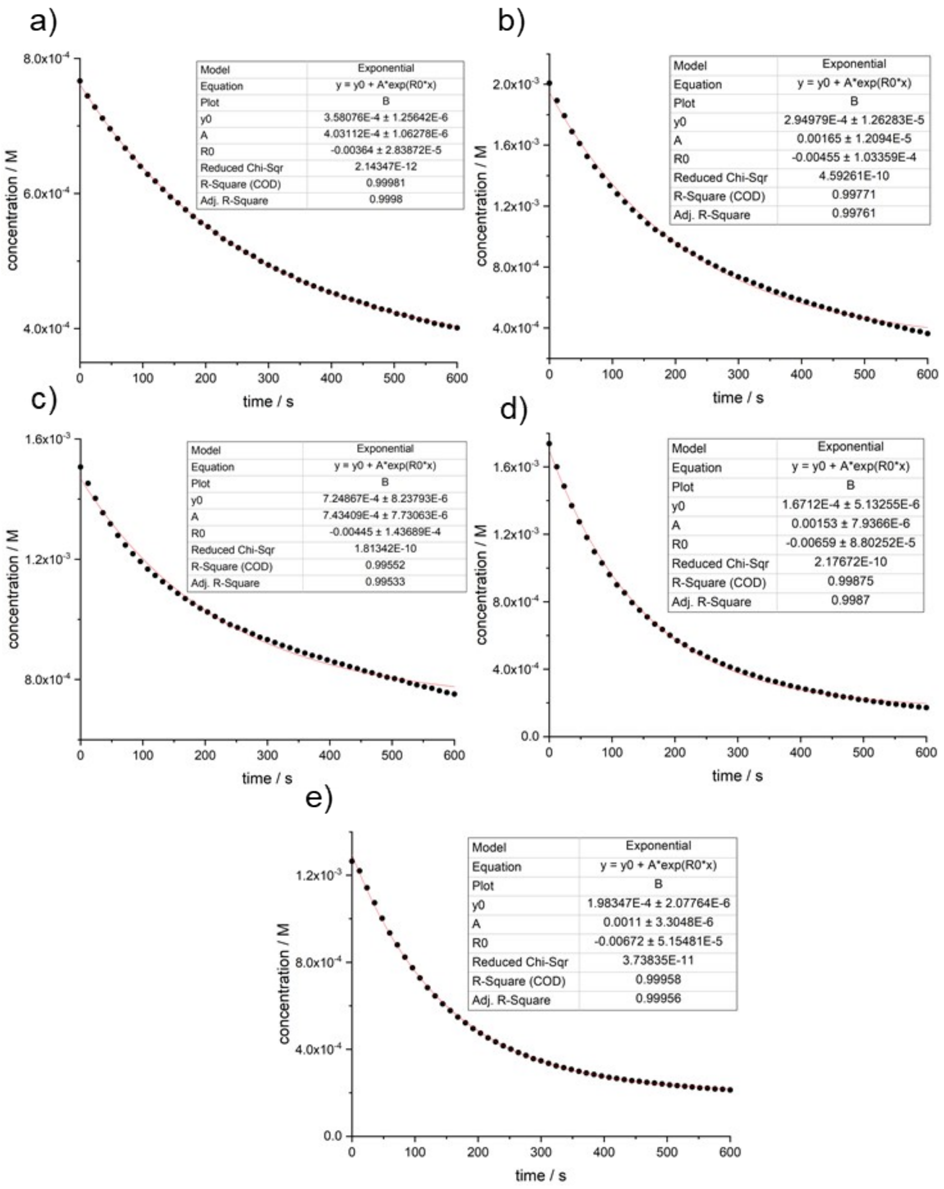

Figure S33. Concentration of a) **4a** (monitored λ = 387 nm), b) **4b** (413 nm), c) **4c** (413 nm), d) **4d** (399 nm), e) **4e** (419 nm) in toluene/MMA 1/1 (v/v) as function of irradiation time (LED385). The red lines indicate the fitting functions and the corresponding parameters are given in the tables.

5. Photo-DSC measurements

The photo-DSC measurements were conducted according to the method previously described by Stueger et al.[5] using a Netzsch DSC 204 F1 with an autosampler. The respective amounts of photoinitiator (0.3 mol% for **4a-e** and Ivocerin®; 0.3 and 0.15 mol% for **5**) were added to the monomer HDDA and mixed in an ultra-sonic bath for 30 min at room temperature. 10 ± 1 mg of each formulation was weighed into an open aluminium pan and all formulations were analyzed in triplicate. The prepared formulations were measured at 25°C under inert atmosphere (N_2-flow = 20 mL min^{-1}). After an equilibration phase of 4 min the samples were irradiated for 6 min with UV light from a Lumen Dynamics OmniCure Series 2001-XLA (320-500 nm). The light intensity was set to 1 W cm-1 and the heat flow of the polymerization reaction was recorded as a function of time. From the theoretical heat of polymerization of the monomer HDDA ($\Delta H_{0,HDDA}$ = 761.92 J g^{-1} or 172.4 kJ mol^{-1}, determined from 86.2 kJ mol^{-1} per acrylate unit[5] the double bond conversion (DBC) was calculated by dividing the measured heat of polymerization ΔH by $\Delta H_{0,HDDA}$. The maximum rate of polymerization $R_{p,max}$ was calculated using equation 1, with h as the height of the exothermic polymerization signal in mW mg^{-1} and ρ = 1010 g L^{-1} as the density of HDDA at 25°C.

$$R_{p,max} = \frac{h \cdot \rho}{\Delta H_{0,HDDA}} \quad \text{(equation 1)}$$

Table S2. Photo-polymerization performance of Ivocerin, tetra(o-toluoyl)germane **(5)** and **4a-e** with HDDA acquired from photo-DSC measurements (time to peak maximum t_{max}, time to 95% of final conversion $t_{95\%}$, maximum rate of photopolymerization $R_{p,max}$ and double bond conversion DBC).

	PI[a] [mol%]	t_{max} [s]	$R_{p,max}$ [mol L^{-1} s^{-1}]	$t_{95\%}$ [s]	DBC [%]
**Ivocerin**	0.3	1.90 ± 0.03	0.30 ± 0.002	20.8 ± 0.9	52.7 ± 3.1
**5**	0.3	2.00 ± 0.06	0.29 ± 0.04	24.8 ± 2.7	56.9 ± 1.4
	0.15	1.90 ± 0.03	0.28 ± 0.002	24.0 ± 1.2	54.1 ± 0.8
**4a**	0.3	1.82 ± 0.27	0.28 ± 0.02	25.9 ± 0.6	55.1 ± 2.1
**4b**	0.3	2.01 ± 0.09	0.21 ± 0.12	21.3 ± 0.6	55.1 ± 0.9
**4c**	0.3	2.00 ± 0.10	0.27 ± 0.01	29.5 ± 0.8	54.5 ± 0.7
**4d**	0.3	1.92 ± 0.05	0.29 ± 0.01	20.2 ± 0.7	55.2 ± 0.9
**4e**	0.3	2.20 ± 0.10	0.27 ± 0.01	21.6 ± 0.5	52.7 ± 1.3

6. DFT Calculations

Computational Methods. The computational studies were executed with the program ORCA4.2.1.[8] Density functional theory (DFT) was used to calculate the geometries and UV-Vis absorption spectra. The simulations were performed in $CHCl_3$ applying the solvation conductor-polarizable continuum model (CPCM).[9] The B3LYP functional[10] supplemented by Grimme's original D3 dispersion correction[11] was applied. Experimental x-ray structures were used as the starting geometries and the def2-SVP basis set[12] was used in the optimization of geometries in combination with the "tight" optimization criteria. The minima on the potential energy surface were confirmed by computing the harmonic frequencies. Vertical excitations were computed by using the def2-TZVP basis set[13] and applying time-dependent density functional theory[14] (TDDFT) calculations with 14 states. The UV spectra were simulated with a half width at half height of 0.1 eV with the program Gabedit.[15]

Table S3. Wavelength, oscillator strength and orbital contributions of the individual vertical excitations S1-S4 for the molecules **4a-4e** as computed with the B3LYP-D3/def2-TZVP/CPCM(CHCl3)//def2-SVP method. Orbital contributions are printed for $c^2 > 0.15$. HOMO abbreviated as H, LUMO abbreviated as L.

		**4a**	**4b**	**4c**	**4d**	**4e**
S1	λ / nm	402.4	430.8	444.3	432.1	447.4
	f	0.0094	0.0040	0.0070	0.0050	0.0096
	Orbitals (c^2)	H→L (0.74)	H→L (0.68)	H→L (0.87)	H→L (0.79)	H-1→L (0.38), H→L (0.23)
S2	λ / nm	384.7	418.4	340.0	400.8	431.4
	f	0.0206	0.0058	0.0058	0.0062	0.0063
	Orbitals (c^2)	H→L+1 (0.67)	H-1→L (0.16), H→L+1 (0.62)	H→L+1 (0.80)	H→L+1 (0.59), H-7→L (0.17)	H-1→L+1 (0.29), H→L+1 (0.29)
S3	λ / nm	344.7	377.5	348.4	357.3	443.8
	f	0.0584	0.0046	0.0072	1.97 E-05	0.0024
	Orbitals (c^2)	H-1→L (0.54)	H-1→L (0.64), H→L+1 (0.19)	H-1→L (0.79)	H-1→L (0.72), H→L (0.22)	H→L (0.65), H-3→L (0.16)
S4	λ / nm	335.2	357.9	345.4	362.5	411.2
	f	0.0144	0.0231	0.0015	0.0013	0.0189
	Orbitals (c^2)	H-1→L (0.17), H-1→L+1 (0.53)	H-1→L+1 (0.70)	H-1→L+1 (0.74)	H-3→L (0.26) H-2→L (0.59)	H-3→L (0.42), H-1→L (0.40)

Table S4. Orbital Energies (in eV) for the molecules **4a-4e** as computed with the B3LYP-D3/def2-TZVP/CPCM(CHCl3)//def2-SVP method. The arrows correspond to orbital energies / H-L gaps that are raised (↑) or lowered (↓) compared to **4b** as the reference.

Molecule	R =	HOMO-1	HOMO	LUMO	LUMO+1	H-L gap
**4a**	Mes	-6.18	↑↑ -5.89	↑↑ -1.66	-1.51	↑↑ 4.23
**4b**	C_6H_5	-6.31	-6.12	-2.11	-1.95	4.01
**4c**	*o*-toloyl	-6.38	↑ -5.96	↑ -1.99	-1.91	↓ 3.97
**4d**	*o*-methoxy	-6.26	↑ -5.95	↑ -1.98	-1.65	↓ 3.97
**4e**	thiofuran	-6.26	↓ -6.20	↓ -2.44	-2.26	↓↓ 3.76

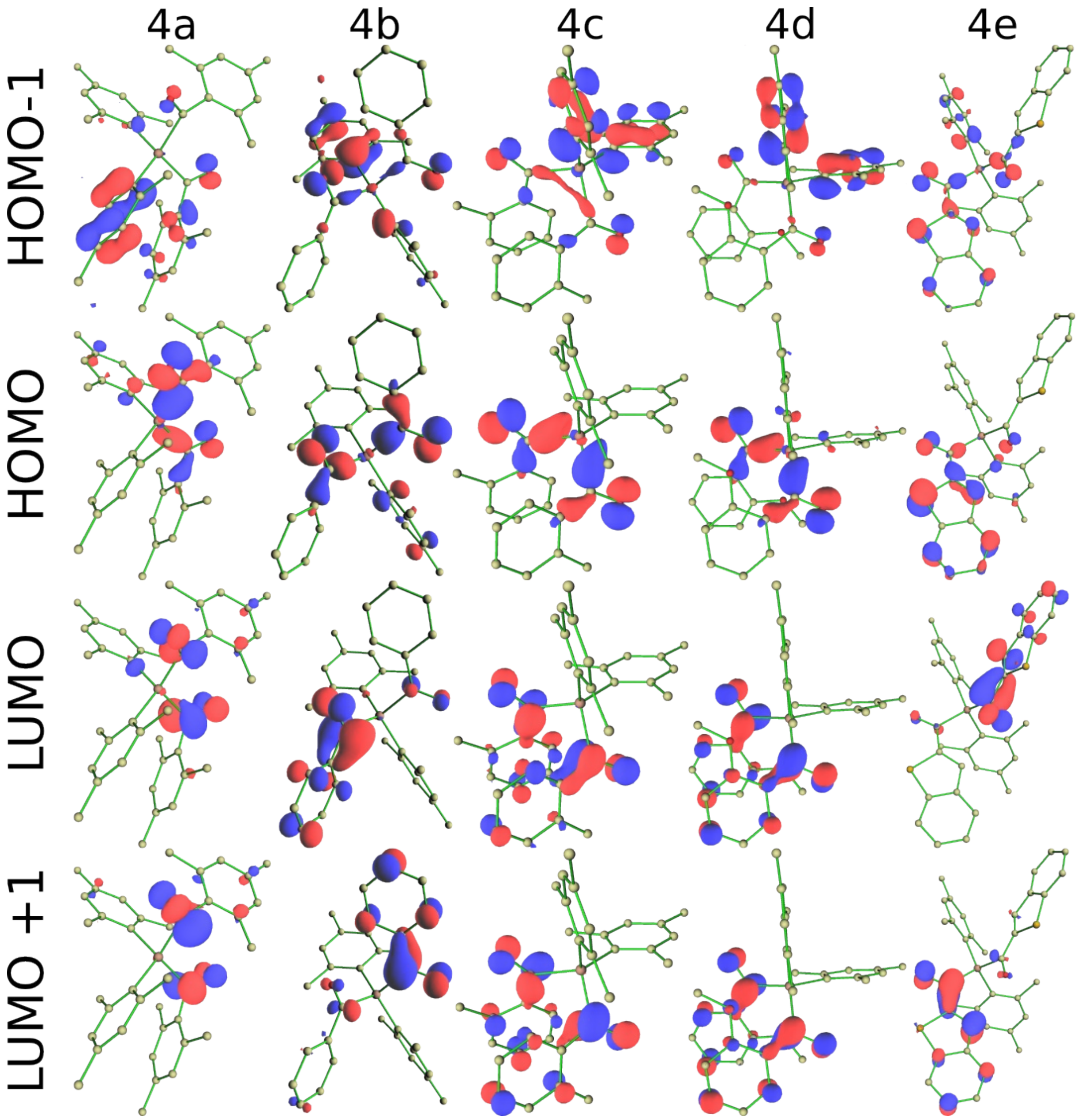

Figure S34. Molecular orbitals HOMO-1, HOMO, LUMO, LUMO+1 for the molecules **4a-4e**. The isovalue is 0.06 a.u.

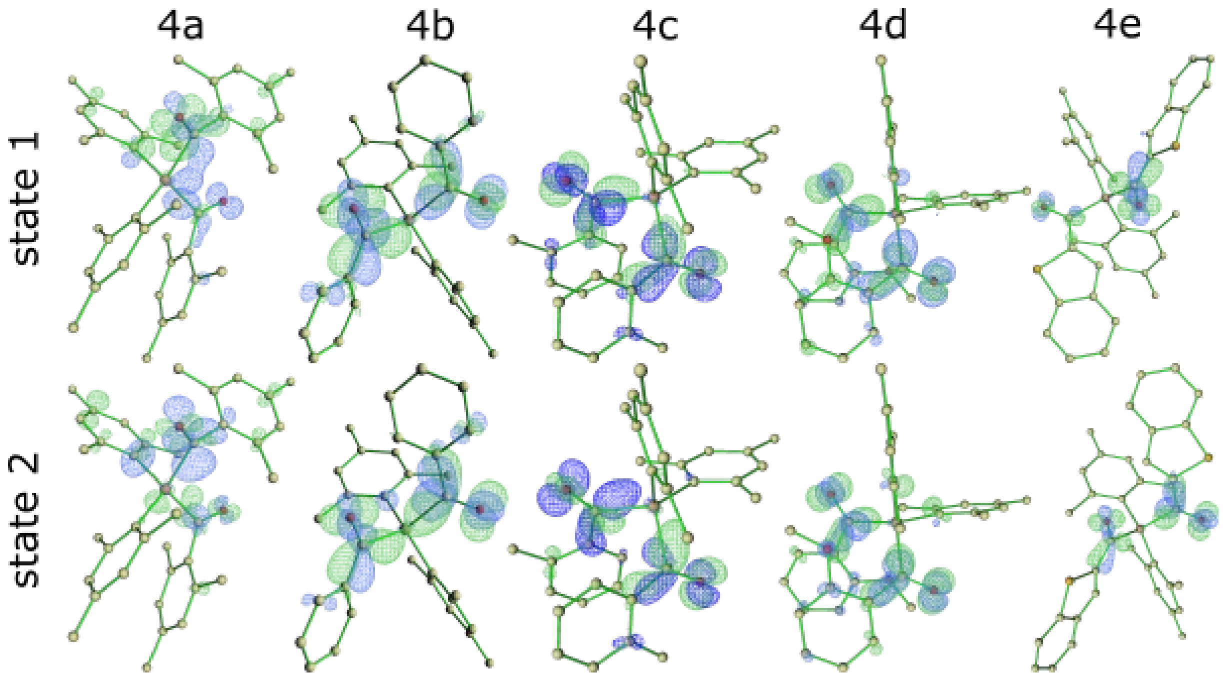

Figure S35. Transition densities for the molecules **4a-4e**. The isovalue is 0.002 a.u.

7. References

[1] A. B. Pangborn, M. A. Giardello, R. H. Grubbs, R. K. Rosen, F. J. Timmers, *Organometallics* **1996**, *15*, 1518.

[2] B. Baasner, J. L. Adcock, J. Houben, E. Müller, T. Weyl, H. Kropf, K. H. Büchel, O. Bayer (Eds.) *Methoden der organischen Chemie*, Thieme, Stuttgart, **1999**.

[3] T. Völkel, Scientific Documentation Bluephase® Family – LED for Every Use, Ivoclar Vivadent AG, Schaan, Liechtenstein, **2009**.

[4] E. Stadler, A. Eibel, D. Fast, H. Freißmuth, C. Holly, M. Wiech, N. Moszner, G. Gescheidt, *Photochem. Photobiol. Sci.* **2018**, *17*, 660.

[5] a)J. Radebner, A. Eibel, M. Leypold, C. Gorsche, L. Schuh, R. Fischer, A. Torvisco, D. Neshchadin, R. Geier, N. Moszner et al., *Angew. Chem. Int. Ed.* **2017**, *56*, 3103, b) J. E. Moore: "Calorimetric Analysis of UV Curable Systems", in ''UV Curing: Science and Technology'', edited by J. E. Moore, Vol. 1 (Technology Marketing Corp.,Stamford, CT, 1978) p. 133

[6] K. M. Baines, W. G. Stibbs, *Coord. Chem. Rev.* **1995**, *145*, 157.

[7] M. Palusiak, S. Simon, M. Solà, *J. Org. Chem.* **2006**, *71*, 5241.

[8] F. Neese, *WIREs Comput Mol Sci* **2012**, *2*, 73.

[9] M. Cossi, N. Rega, G. Scalmani, V. Barone, *J. Chem. Phys.* **2001**, *114*, 5691.

[10] a) A. D. Becke, *J. Chem. Phys.* **1993**, *98*, 5648; b) P. J. Stephens, F. J. Devlin, C. F. Chabalowski, M. J. Frisch, *J. Phys. Chem.* **1994**, *98*, 11623.

[11] S. Grimme, J. Antony, S. Ehrlich, H. Krieg, *J. Chem. Phys.* **2010**, *132*, 154104.

[12] A. Schäfer, H. Horn, R. Ahlrichs, *J. Chem. Phys.* **1992**, *97*, 2571.

[13] F. Weigend, R. Ahlrichs, *Phys. Chem. Chem. Phys.* **2005**, *7*, 3297.

[14] F. Weigend, M. Häser, H. Patzelt, R. Ahlrichs, *Chem. Phys. Let.* **1998**, *294*, 143.

[15] A.-R. Allouche, *J. Comput. Chem.* **2011**, *32*, 174.
